# High-Throughput Identification of Promoters and Screening of Highly Active Promoter-5′-UTR DNA Region with Different Characteristics from *Bacillus thuringiensis*

**DOI:** 10.1371/journal.pone.0062960

**Published:** 2013-05-10

**Authors:** Jieping Wang, Xulu Ai, Han Mei, Yang Fu, Bo Chen, Ziniu Yu, Jin He

**Affiliations:** State Key Laboratory of Agricultural Microbiology, College of Life Science and Technology, Huazhong Agricultural University, Wuhan, Hubei, People’s Republic of China; Indian Institute of Science, India

## Abstract

In bacteria, both promoters and 5′-untranslated regions (5′-UTRs) of mRNAs play vital regulatory roles in gene expression. In this study, we identified 1203 active promoter candidates in *Bacillus thuringiensis* through analysis of the genome-wide TSSs based on the transcriptome data. There were 11 types of σ-factor and 34 types of transcription factor binding sites found in 723 and 1097 active promoter candidates, respectively. Moreover, within the 1203 transcriptional units (TUs), most (52%) of the 5′-UTRs were 10–50 nucleotides in length, 12.8% of the TUs had a long 5′-UTR greater than 100 nucleotides in length, and 16.3% of the TUs were leaderless. We then selected 20 active promoter candidates combined with the corresponding 5′-UTR DNA regions to screen the highly active promoter-5′-UTR DNA region complexes with different characteristics. Our results demonstrate that among the 20 selected complexes, six were able to exert their functions throughout the life cycle, six were specifically induced during the early-stationary phase, and four were specifically activated during the mid-stationary phase. We found a direct corresponding relationship between σ-factor-recognized consensus sequences and complex activity features: the great majority of complexes acting throughout the life cycle possess σ^A^-like consensus sequences; the maximum activities of the σ^F^-, σ^E^-, σ^G^-, and σ^K^-dependent complexes appeared at 10, 14, 16, and 22 h under our experimental conditions, respectively. In particular, complex P*hj3* exhibited the strongest activity. Several lines of evidence showed that complex P*hj3* possessed three independent promoter regions located at −251∼−98, −113∼−31, and −54∼+14, and that the 5′-UTR +1∼+118 DNA region might be particularly beneficial to both the stability and translation of its downstream mRNA. Moreover, P*hj3* successfully overexpressed the active β-galactosidase and turbo-RFP, indicating that P*hj3* could be a proper regulatory element for overexpression of proteins in *B*. *thuringiensis*. Therefore, our efforts contribute to molecular biology research and the biotechnological application of *B. thuringiensis*.

## Introduction

Unlike archaea and eukaryotes, bacteria contain only one form of RNA polymerase (RNAP) core enzyme comprised of five subunits (α2ββ′ω). However, bacteria possess multiple forms of a specific σ subunit (σ-factor) and thus multiple forms of RNAP holoenzymes, which, in turn, bind to their cognate promoters to initiate transcription of specific genes (or operons) [1]–[4]. In bacteria, a promoter is a specific DNA sequence that provides secure initial binding sites for RNAP to initiate transcription of a particular gene (or operon) [1], [2]. The core promoter includes a transcription start site (TSS) and two hexameric elements centered at or near –10 and –35 positions relative to the TSS. Some promoters contain one or more upstream promoter (UP) elements and the TGn extended –10 element, among others [1]–[4].

A TSS is an important marker of an active promoter, and mapping the TSSs is therefore a novel and effective strategy for the identification of active promoters. McGrath et al. mapped 769 TSSs and subsequently identified 27 promoter motifs in *Caulobacter crescentus* using a high-density array that was specifically designed to detect the TSS positions [5]. Mendoza-Vargas et al. mapped more than 1700 TSSs and identified a large number of promoters that control the expression of approximately 800 genes in *Escherichia coli* by combining a modified 5′ RACE protocol and an unbiased high-throughput pyrosequencing strategy [6]. However, the active promoter candidates acquired by them were not verified through further experimentation. Recently, the high-throughput and unbiased sequencing of the cDNA (RNA-seq) technique has been used for whole-genome transcriptomics analyses of diverse bacteria [7]. Sharma et al. reported that the genome-wide TSSs could be directly detected from RNA-seq data using a novel differential approach selective for the 5′ triphosphate (5′-PPP) ends of the primary transcripts [8]. Although Sharma et al. did not report the data of active promoter identification, the knowledge of TSSs could provide us with a promising opportunity for the high-throughput identification of active promoters from RNA-seq data.

Besides the promoters, the 5′-untranslated regions (5′-UTRs) of bacterial mRNA are also known to play important regulatory roles in gene expression, which possibly occur at the transcriptional, post-transcriptional, or translational levels [9]. Extremely diverse mechanisms are employed by the *cis*-acting RNA regulatory elements in 5′-UTRs to strictly adjust the cellular levels of their downstream genes, including: (i) the ability of many 5′-UTRs to recognize a specific regulatory signal, such as T-boxes, riboswitches and RNA thermometers [10]–[12]; (ii) the capability of some 5′-UTRs to provide binding sites for small regulatory RNAs [9], [13]; and (iii) more 5′-UTRs being able to regulate the expression of the downstream gene, presumably by RNase III-mediated cleavage modification [14], preventing degradation of the mRNA [15], or other unknown mechanisms. Therefore, besides promoters, some 5′-UTR DNA regions have a significant applied potential in molecular biology research and improvement of recombinant protein expression [9], [12], [16], [17].

*Bacillus thuringiensis* is characterized by the formation of parasporal crystals consisting of insecticidal crystal proteins (ICPs) during sporulation. Moreover, the accumulation of ICPs can account for 20–30% of the cell’s dry weight [18]. This unique advantage enables *B. thuringiensis* to be not only the most widely used environmentally compatible biopesticide [19], [20] but also a promising gene expression system. In the *Bacillus* species, the sporulation-specific σ-factors SigH, SigF, SigE, SigG, and SigK are spatially and temporally activated to control the process of sporulation [21]. SigF and SigE regulate early compartmentalized gene expression, whereas SigG and SigK activate transcription of the genes that build the structural components of the spore [21]–[23]. SigE and SigK also promote transcription of the ICP genes for the formation of parasporal crystals in *B*. *thuringiensis* [24]. Consequently, to thoroughly investigate the regulation of gene expression and/or construct a novel gene expression system in *B. thuringiensis*, high-throughput identification and screening of promoter-5′-UTR DNA region complexes (to avoid redundancy, “complex” refers to the promoter region and the 5′-UTR DNA region) with specific characteristics (intrinsic strength and temporal activation) are of great practical significance.

*B. thuringiensis* subsp. *chinensis* CT-43 is the first sequenced strain harboring ICP genes [25]. Moreover, the whole-genome transcriptomics analysis of CT-43 at four different growth phases in GYS medium [26] was performed by the RNA-seq technique. In the RNA-seq data, the average length of the clean-reads was 110 nucleotides, and the number of the clean-reads in the four different libraries was 577,810 to 1,493,721. Thus, the sequencing coverage of the four growth phases was 10- to 27-fold. Moreover, the percentages of the clean-reads that were mapped to the CT-43 genome were approximately 90 to 96% [27]. In this study, 1203 active promoter candidates were identified from the RNA-seq data, and 20 highly active promoter candidates combined with the corresponding 5′-UTRs were selected to perform further analyses to screen the highly active promoter-5′-UTR DNA region complexes with different characteristics.

## Materials and Methods

### Bacterial Strain and Plasmids

The bacterial strains and plasmids used in this study are listed in Table S1.

### Genome-wide TSS Mapping and Identification of Active Promoter Candidates

Using RNA-seq method, we previously acquired transcriptome data of *B*. *thuringiensis* strain CT-43 at four growth phases when grown in GYS medium [26] at 28°C and 200 rpm: 7 h (the mid-exponential growth phase), 9 h (the early-stationary growth phase), 13 h (the mid-stationary growth phase, sporulation), and 22 h (the spore maturation and mother cell lysis phase) [27]. To map genome-wide TSSs, the clean-reads of each sample were mapped to the CT-43 genome using BlastN with a threshold *e* value of 0.00001 and the “−F F” parameter [28], and then the number of unambiguously mapped reads per nucleotide was calculated and visualized by R and Origin version 8.0. According to the mapping data, all 5′-ends that showed obvious cDNA coverage enrichment were annotated to predict the TSSs.

The regions located ≤500 nucleotides upstream of the mapped TSS were taken as the active promoter candidates. Then, these 500-nucleotide sequences were submitted to DBTBS [29] (http://dbtbs.hgc.jp/) to identify the recognition sites for σ-factors and transcription factors (TFs) through “Weight Matrix Search (by sequence)”. During the “advanced search”, the threshold of the *p*-value was set as 0.05.

### Construction of Plasmids

All promoter-5′-UTR DNA region complexes were designated as P*hj* with the corresponding serial numbers.

#### Construction of translational fusion plasmids

All primers used in this study are listed in Table S2. The translational fusion plasmid pHT1K-P*hj1*-*lacZ* was constructed through the experimental procedure shown in Figure S1. Briefly, the promoter-5′-UTR DNA region complex of P*hj1* was amplified from the genomic DNA of CT-43 using the primer pair P*hj1*-F/P*hj1*-R that carried additional recognition sites of the restriction endonucleases *Nco*I, *Xba*I and *Not*I at the 5′-end and *Bam*HI and *Sma*I at the 3′-end. The PCR products were digested and ligated with the shuttle plasmid pHT1K [30] at the 5′ *Bgl*II and 3′ *Pst*I restriction sites and then transformed into *E. coli* strain DH5α to construct the plasmid pHT1K-P*hj1*. The *lacZ* gene without the 5′-UTR DNA region was amplified from the plasmid pHT304-18Z [31]. The amplified products were digested with *Bam*HI and *Kpn*I, inserted into the plasmid pHT1K-P*hj1* and then transformed into *E. coli* DH5α to acquire the plasmid pHT1K-P*hj1*-*lacZ*. All other translational fusion plasmids were obtained by replacing P*hj1* with amplified promoter-5′-UTR DNA region complexes at 5′ *Nco*I and 3′ *Bam*HI sites (Figure S1).

#### Construction of transcriptional fusion plasmids using fragments from Phj3

To analyze the characteristics of complex P*hj3* in detail, the *lacZ* gene with its 5′-UTR DNA region was digested with *Bam*HI and *Kpn*I from the plasmid pHT304-18Z and inserted into the plasmid pHT1K to obtain the plasmid pHT1K-*lacZ*(UTR). Seven fragments of complex P*hj3*, including −251∼−98, −251∼−31, −251∼+14, −113∼−31, −54∼+14, −54∼+118, and −6∼+118 were amplified with the cognate primer pairs (Table S1). Subsequently, the PCR products of the seven fragments were separately digested with *Nco*I and *Bam*HI and inserted into the plasmid pHT1K-*lacZ*(UTR) to construct the corresponding transcriptional fusion plasmids.

#### Construction of chimeric complexes

The 5′-UTR DNA fragment +1∼ +118 of complex P*hj3* was separately fused at the 3′-ends of the promoter regions of complexes P*hj12* and P*hj17* to construct the chimeric complexes named as cP*hj12* and cP*hj17* by overlapping PCR. Next, the PCR products were used to replace P*hj1* in the plasmid pHT1K-P*hj1*-*lacZ* to acquire the translational fusion plasmids pHT1K-cP*hj12*-*lacZ* and pHT1K-cP*hj17*-*lacZ* (see Figure S1).

#### Construction of plasmids for protein overexpression

The *turbo-rfp* gene was amplified by PCR using *rfp*-F/*rfp*-R as the primers and the plasmid pRP1028 (a gift from Scott Stibitz, Center for Biologics Evaluation and Research, Food and Drug Administration, Bethesda, Maryland, USA) as the template. The amplified products were digested with *Bam*HI and *Kpn*I and inserted into the plasmid pHT1K-P*hj3* to construct the plasmid pHT1K-P*hj3*-*turbo*-*rfp* (Figure S2).

### Transformation of the Plasmids to *B. thuringiensis* BMB171

After confirmation by sequencing, the plasmids were extracted from *E. coli* DH5α and transformed (electroporation) into *B. thuringiensis* BMB171 [32]. Various transformants were harvested by screening the clones in LB plates with 25 µg/mL erythromycin. Here, each transformant was not designated as a new strain, but rather expressed as BMB171 containing a specific plasmid.

### Determination of β-Galactosidase Activity

The *B*. *thuringiensis* strain BMB171 containing each translational fusion plasmid or transcriptional fusion plasmid with the *lacZ* reporter gene was grown at 28°C in an orbital shaker at 200 rpm in GYS medium with 25 µg/mL erythromycin. Samples were taken at 2 h intervals for the determination of β-galactosidase activities. The growth curve was obtained by determining the optical density (OD) at 600 nm (OD_600_) combined with observation under a phase contrast microscope (Nikon ECLIPSE E6000, Nikon Corp., Tokyo, Japan). The β-galactosidase specific activities were determined and converted to Miller units as previously described [33]. The values shown represent the average of three independent experiments.

### SDS-PAGE Analysis of Overexpressed Proteins

Each recombinant BMB171 strain containing pHT1K-P*hj3*-*lacZ* or pHT1K-P*hj3*-*turbo*-*rfp* plasmid was grown at 28°C for 22 h in LB medium with 25 µg/mL erythromycin. The culture was harvested by centrifugation and the crude proteins were extracted by boiling. SDS-PAGE was performed with 5% (w/v) stacking gels and 12% (w/v) separating gels, and proteins were visualized by Coomassie Blue R-250 staining.

### Accession Number

The RNA-seq data from this article are available as raw short read data in the NCBI’s GEO database under accession number GSE39479.

## Results

### Identification of Active Promoter Candidates from RNA-seq Data

#### Genome-wide TSS mapping

After calculating the number of unambiguously mapped reads per nucleotide, we observed the cDNA coverage enrichment at all 5′-ends of the highly expressed genes that showed high redundancy in RNA-seq data. Generally, a TSS is manually determined once (i) a substantially sharp cDNA coverage enrichment is observed at the 5′-end, or (ii) a sharp cDNA coverage enrichment at the 5′-end appears in at least two libraries of the four growth phases [8], [34]; the TSSs of the remaining genes with low expression levels were unable to be unambiguously determined due to the relatively low signal-to-noise ratio. Following this principle, 1203 TSSs were mapped in the CT-43 genome, of which 1125 and 78 TSSs were shared by chromosome and plasmids, respectively (Table S3). Interestingly, 76 genes located within specific operons were found to have their own TSSs, such as the gene CT-43_CH1330 (indicated as “operon (intra)” in Table S3). Figure S3 shows the substantially sharp cDNA coverage enrichment at TSS positions of the 20 complex candidates P*hj1*-P*hj20*, which were selected for further analyses in this study.

#### Prediction of σ-factor and TF binding sites

The mapped 1203 TSSs represented 1203 active promoter candidates. To analyze the putative binding sites for σ-factors and TFs, 500-nucleotide sequences located upstream of the mapped TSSs were submitted one by one to DBTBS [29] (http://dbtbs.hgc.jp/). Using the “Weight Matrix Search (by sequence)” with the threshold set at a *p*-value 0.05, we identified the putative binding sites for SigA (209, 17.4%), SigB (78, 6.5%), SigD (26, 2.2%), SigE (129, 10.7%), SigF (105, 8.7%), SigG (112, 9.3%), SigH (190, 15.8%), SigK (72, 6.0%), SigL (22, 1.8%), SigW (49, 4.1%), and SigX (25, 2.1%). However, the putative σ-factor binding sites of 480 (about 40%) active promoter candidates could not be predicted (Table S3). Among the 723 active promoter candidates that could be predicted to possess the putative σ-factor binding sites, 495 (68.5%) were possibly controlled by a single σ-factor, while 228 (31.5%) were possibly controlled by multiple σ-factors. It is worth mentioning that 491 (68.0%) promoters were found to possess the putative binding sites for the sporulation-specific σ-factors SigH, SigF, SigE, SigG, and SigK (Table S3), reflecting that transcription of the corresponding genes was temporally activated during sporulation.

There were 34 different TF binding sites found in 1097 active promoter candidates (Table S3). The most frequently found TF binding sites were those for DegU (437), ComK (267), PerR (217), CodY (196), Fur (150), AbrB (125), AhrC (125), Zur (119), PurR (106), and ResD (101) (Table S3). These results indicated that a complicated TF regulatory network was involved in gene expression in *B*. *thuringiensis*, and that the TFs DegU, ComK, PerR, CodY, Fur, AbrB, AhrC, Zur, PurR, and ResD played more important roles than the others under our experimental conditions.

#### Length of the 5′-UTRs

In terms of the 5′-UTR length (ranging from the TSS to the first annotated start codon ATG of the corresponding DNA rigion) for the 1203 transcriptional units (TUs), we found that: i) most (52.0%) of the 5′-UTRs were 10–50 nucleotides in length; ii) the length of 18.9% 5′-UTRs varied between 50 and 100 nucleotides; iii) 12.8% of TUs had a long 5′-UTR (between 100 and 350 nucleotides in our data); and iv) 16.3% of TUs were leaderless (typically, a mRNA is considered as “leaderless” if the length of 5′-UTRs is less than ten nucleotides [8]) (Figure S4 and Table S3). In addition, the TSS of the gene pCT127.010 is located two nucleotides downstream of the first annotated ATG codon, perhaps owing to an error annotation. For the 5′-UTRs that were longer than 50 nucleotides, we searched them in the Rfam database [35] to identify known regulatory RNA elements. We found that five TUs most likely have an RNA regulatory element, including the CH1169 gene (T-box), *rplS* operon (L19_leader), *rplU* operon (L21_leader), *infC* operon (L20_leader), and CH5446 (SAM-riboswitch).

Using *lacZ* as a reporter gene, 20 active promoter candidates together with their corresponding 5′-UTR DNA rigions (promoter-5′-UTR DNA region complexes**)** were selected to further investigate their activity features, including intrinsic strength, temporal activation, and the consensus sequences recognized by σ-factor (Tables S4 and S5). According to the RNA-seq data, nine complex candidates could be able to exert their functions throughout the life cycle, seven could be specifically induced in the early-stationary phase and four could be specifically activated in the mid-stationary phase.

### The Life Cycle of Strain BMB171 in GYS Medium

**Figure 1:**
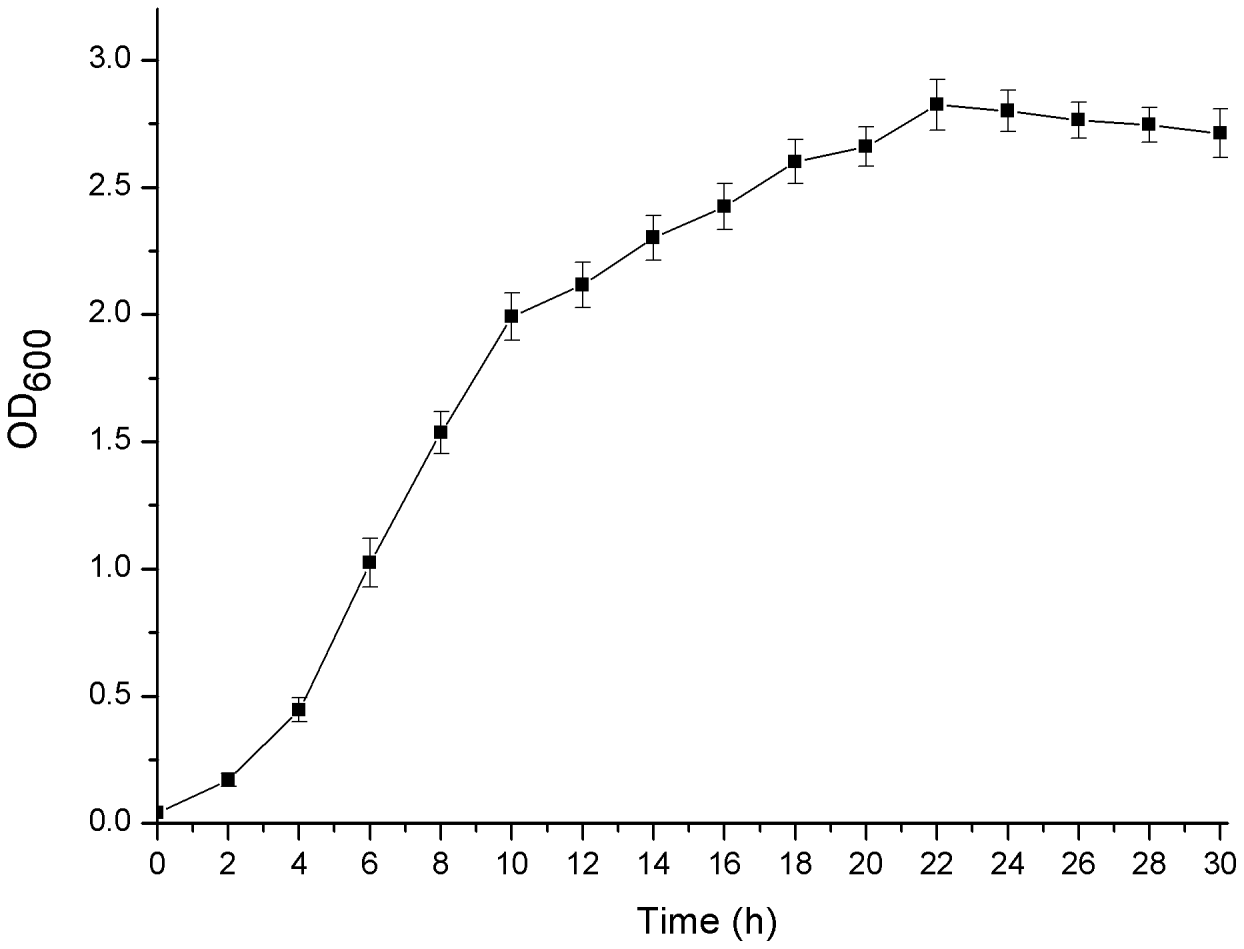
Growth curve of strain BMB171 containing the control plasmid pHT1K in GYS medium. The strain BMB171 containing the control plasmid pHT1K was grown in GYS medium with 25 µg/mL erythromycin. The *y*-axis presents the average optical densities of triplicate bacterial cultures at 600 nm at each time point. Data are averages of three independent experiments (error bars are SEM from mean values).

The life cycle of *B. thuringiensis* can be differentiated into two distinctively different stages: vegetative growth and sporulation. Because various σ-factors are temporally and/or spatially activated at different growth phages to control the process of vegetative growth and sporulation [21]–[23], the determination of the life cycle is necessary to analyze the features of the complexes with specific characteristics. By measuring the OD_600_, a growth curve of strain BMB171 containing the control plasmid pHT1K in GYS medium with 25 µg/mL erythromycin was obtained (Figure 1). These results combined with the obervation under a phase contrast microscope indicated that: 1) the growth of strain BMB171 containing pHT1K entered the early-stationary phase after appproximately 10 h of growth and the cells began to aggregate; 2) the 16 h time point represented the mid-stationary phase and the percentage of sporulating cells reached approximately 30%; 3) from approximately 22 h, BMB171 containing pHT1K entered the spore maturation and mother cell lysis phase, and approximately 30% mother cells were lysed with some spore release.

### Screening of the Highly Active Promoter-5′-UTR DNA Region Complexes with Different Characteristics

#### The complexes acting throughout the life cycle

**Figure 2:**
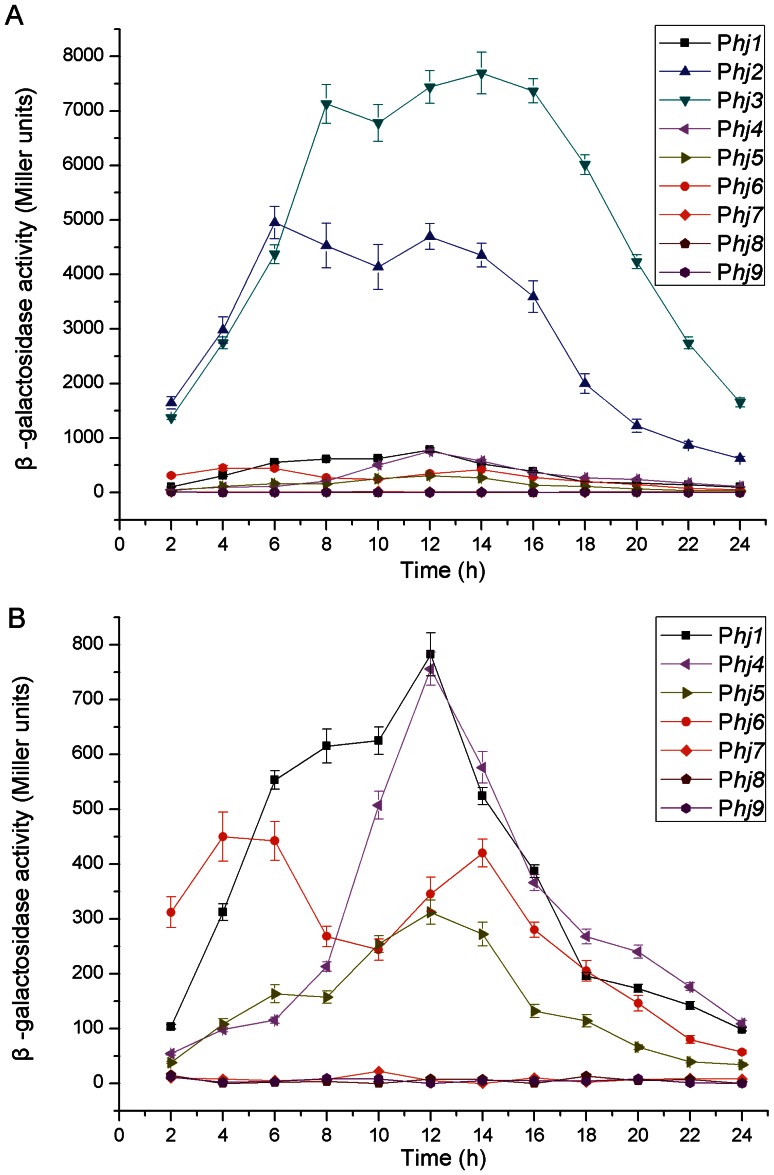
Activity analyses of the complex candidates acting throughout the life cycle. (A) β-galactosidase specific activities directed by complexes P*hj1*-P*hj9*. (B) β-galactosidase specific activities directed by complexes P*hj1*, and P*hj4*-P*hj9.* Data are averages of three independent experiments (error bars are SEM from mean values).

Candidates from P*hj1* to P*hj9* were selected to screen the highly active promoter-5′-UTR DNA region complexes, which can exploit their activity throughout the life cycle (Table S4). Our results showed that complex P*hj3* displayed the strongest activity, followed by P*hj2*, P*hj1,* P*hj4*, and P*hj6* (Figure 2A and Figure 2B). The maximum β-galactosidase specific activities directed by complexes P*hj3* and P*hj2* were approximately 7,600 and 5,000 Miller units in GYS medium, respectively; they reached 11,000 and 8,400 Miller units in LB medium (data not shown), respectively. Moreover, the P*hj3*-directed β-galactosidase activity could be detected at the onset of growth (2 h). It reached the first and second peaks at 8 and 14 h of growth, respectively, and then remained at a high level throughout the life cycle (Figure 2A). Being similar to the promoter of complex P*hj3*, the promoters of P*hj2* and P*hj6* also appeared to exhibit a second induction phenomenon, possibly owing to the fact that these promoters all possess more than one kind of consensus sequences that might be controlled by at least two different σ-factors (Table S5). Unfortunately, the activities of complex candidates P*hj7*, P*hj8*, and P*hj9* from the plasmids of strain CT-43 could not be detected in strain BMB171 (Figure 2B). It is unclear why complex P*hj6* also came from a plasmid of strain CT-43, but it was confirmed to work normally in strain BMB171 (Figure 2B). Thus, the reason for why complex candidates P*hj7*, P*hj8*, and P*hj9* could not exert their functions remains to be elucidated.

#### The complexes specifically induced during the early-stationary phase

**Figure 3:**
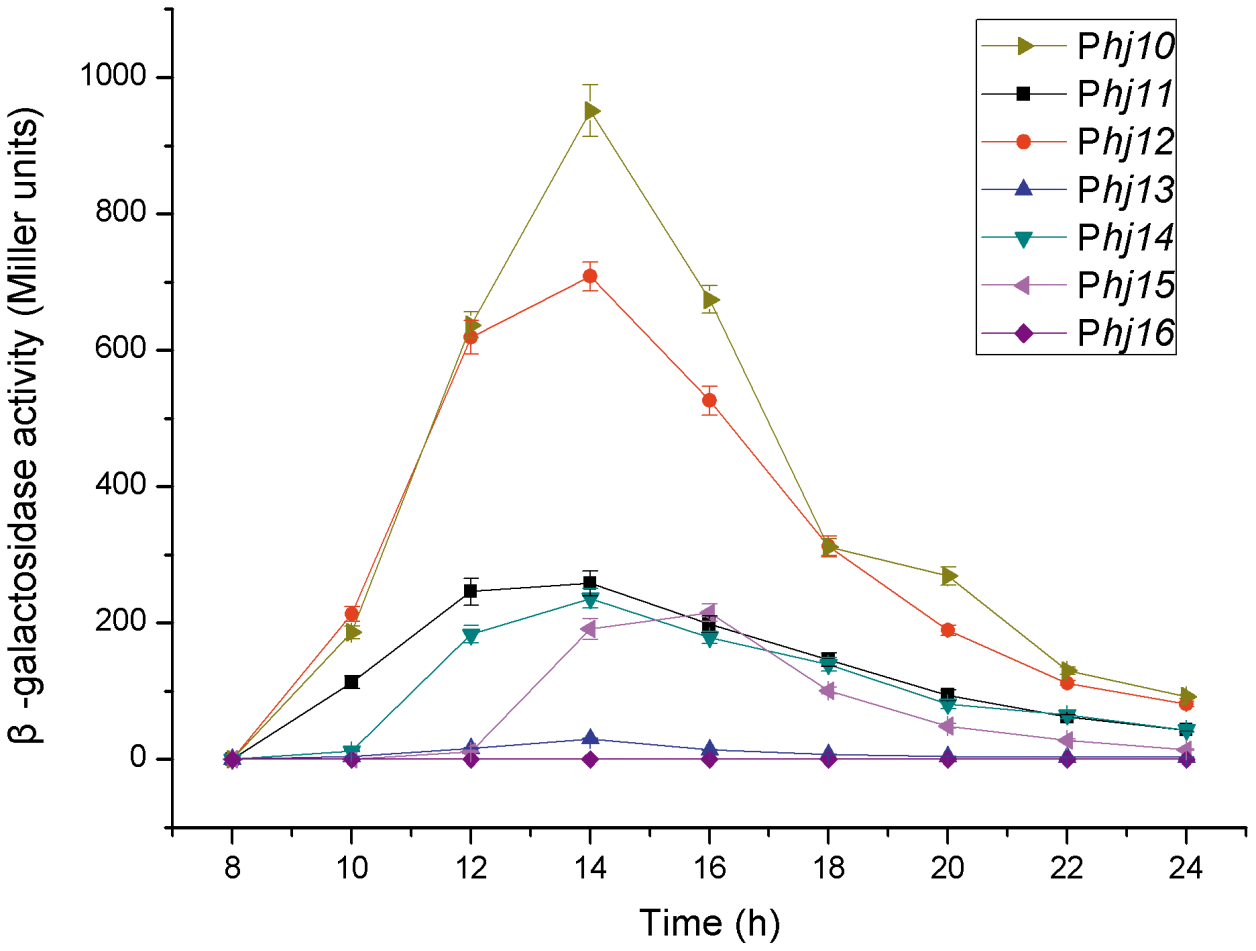
Activity analyses of the complex candidates specifically induced in the early-stationary growth phase. Complexes from P*hj10* to P*hj16* were separately fused with the gene *lacZ* and their activities were monitored by detecting the β-galactosidase specific activities. Data are averages of three independent experiments (error bars are SEM from mean values).

Further analyses were performed on the seven complex candidates P*hj10*-P*hj16* that could specifically exert their functions in the early-stationary phase (Table S4). Our results showed that complex P*hj10* possessed the strongest activity among the seven analyzed complex candidates, followed by complex P*hj12* (Figure 3). Interestingly, β-galactosidase activities directed by complexes P*hj10*, P*hj11*, P*hj12*, and P*hj14*, which have the σ^E^-like consensus sequences (Table S5), all reached the peak values at approximately 14 h (early-stationary phase), whereas the highest activity of complex P*hj15* containing the σ^G^-like consensus sequence appeared 2 h later (at 16 h) compared to the σ^E^-dependent complex (Figure 3). These results truly reflect the temporal regulation of SigE and SigG in *B. thuringiensis*. In addition, the activity of complex P*hj13* was very weak, and that of complex P*hj16* could not be detected.

#### The complexes specifically activated during the mid-stationary phase

**Figure 4:**
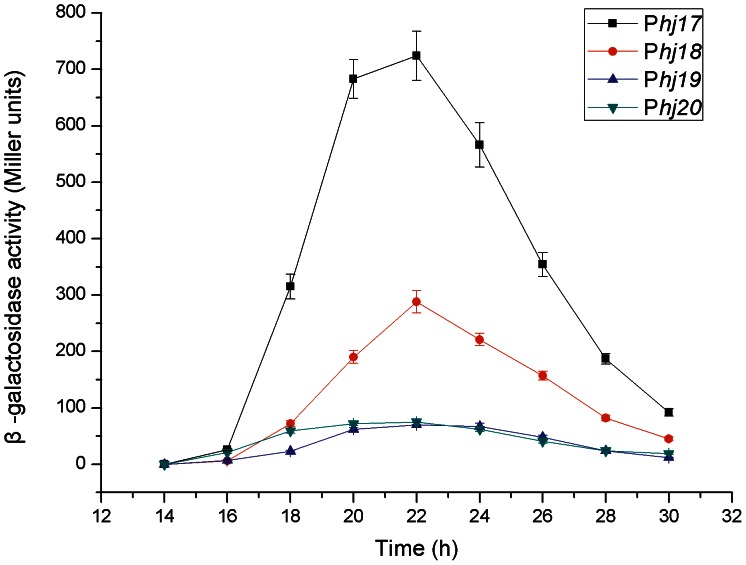
Activity analyses of the complex candidates specifically induced in the mid-stationary growth phase. Complexes P*hj17*, P*hj18*, P*hj19*, and P*hj20* were separately fused with the gene *lacZ* and their activities were monitored by detecting the β-galactosidase specific activities. Data are averages of three independent experiments (error bars are SEM from mean values).

Complex candidates P*hj17*-P*hj20*, which are specifically activated in the mid-stationary phase, were selected to be further confirmed by translational fusion analysis. The results indicated that the analyzed complexes all began induction at approximately 16 h and reached the maximum inductions at 22 h of growth (Figure 4). These results were in excellent agreement with the fact that these complexes all contain the σ^K^-like consensus sequences (Table S4). Among them, complex P*hj17* shared the strongest activity, whereas complexes P*hj19* and P*hj20* had weak activities (Figure 4).

### Characteristics of Complex P*hj3*

**Figure 5:**
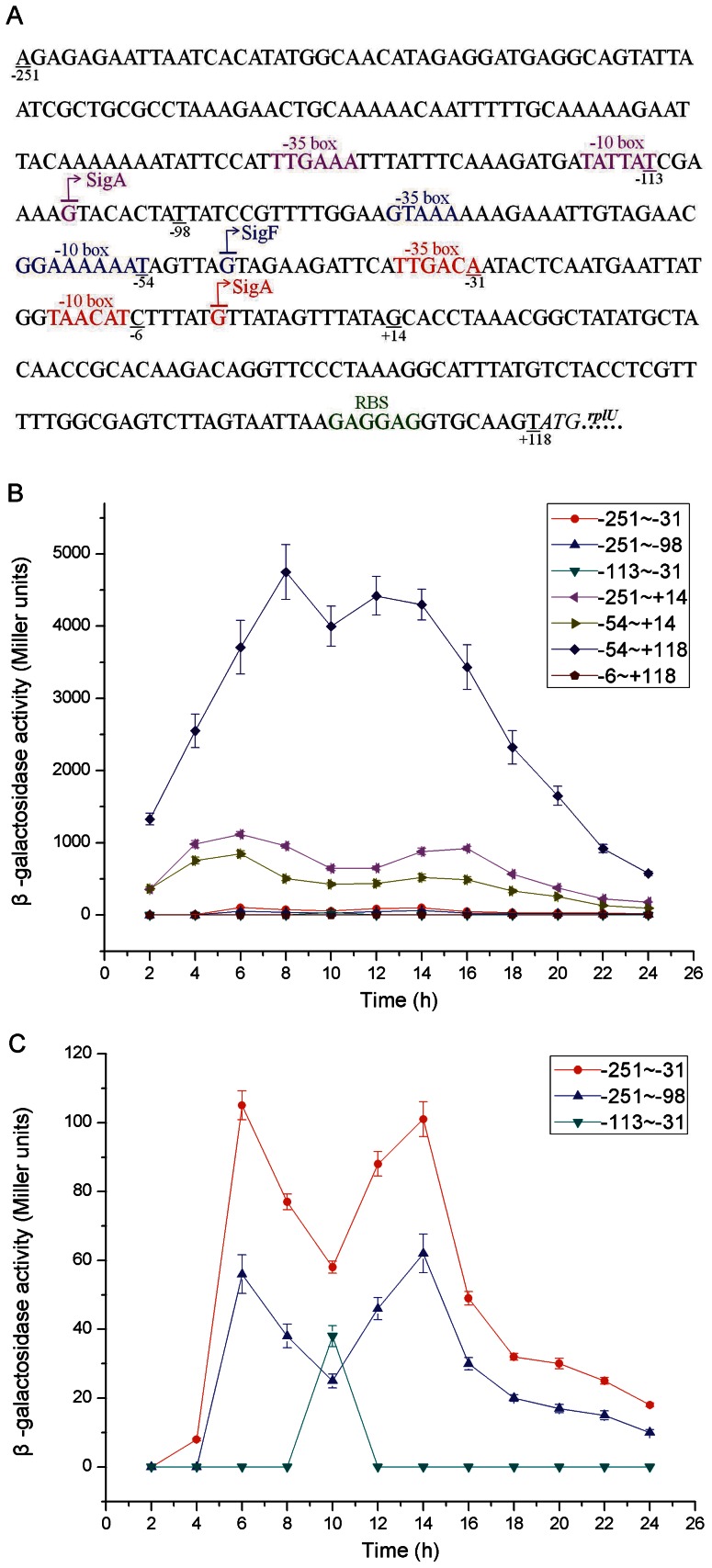
Characteristics of the structure and activity of complex P *hj3*. (A) Nucleotide sequence and structure characteristics of complex P*hj3*. The consensus sequences recognized by σ-factor (σ^A^ and σ^F^) and the corresponding TSSs, as well as the RBS are highlighted in different colors. (B) β-galactosidase specific activities directed by the fragments −251∼−98, −251∼−31, −251∼+14, −113∼−31, −54∼+14, −54∼+118 and −6∼+118 of complex P*hj3*. (C) β-galactosidase specific activities directed by the fragments −251∼−98, −251∼−31, and −113∼−31 of complex P*hj3*. Data are averages of three independent experiments (error bars are SEM from mean values).

Complex P*hj3* was found to share the strongest activity in *B*. *thuringiensis* in this study, and therefore we examined its characteristics in more detail. To perform transcriptional fusion analysis, we divided complex P*hj3* into 7 different fragments: −251∼−98, −251∼−31, −251∼+14, −113∼−31, −54∼+14, −54∼+118 and −6∼+118 (Figure 5A).

The fragments −251∼−31 and −54∼+14 contain the σ^A^-like consensus sequences TTGAAA and TATTAT in the −35 elements, and TTGACA and TAACAT in the −10 elements (Figure 5A and Table S4). The fragment −113∼−31 has the σ^F^-like consensus sequence (Figure 5A and Table S4). The results demonstrated that each of the three fragments (−251∼−31, −113∼−31, and −54∼+14) could act as an independent promoter (Figure 5B and Figure 5C). Among them, the activity of the promoter −113∼−31 was the weakest, and the activity of the promoter −54∼+14 was 14-fold higher than the promoter −251∼−98. Accordingly, the promoter −54∼+14 would be a major contributor to the promoter of complex P*hj3* activity.

The two truncated promoters −251∼−31 and −54∼+14 appeared to have a second induction and exerted their activities throughout the life cycle similar to the full-length promoter. In addition, although the activity of the truncated promoter −113∼−31 was relatively low, it reached the maximum value after 10 h of growth, which was in agreement with the fact that the fragment −113∼−31 contains the σ^F^-like consensus sequences (Figure 5A and 5C).

It is important to note that the β-galactosidase activity directed by fragment −54∼+118 was approximately nine times higher than fragment −54∼+14, but the fragment −6∼+118 did not share the promoter activity (Figure 5B). Accordingly, we hypothesized that the fragment −6∼+118 could play a certain additional regulatory role contributing to the production of β-galactosidase. To investigate this possibility, we examined the RNA secondary structure of the RNA transcript from +1∼+118 through Mfold [36]. Exhilaratingly, the RNA fragment +1∼+118 preferred to fold into a perfect stem-loop structure, and more importantly, the ribosome binding site (RBS) became accessible due to its localization on the loop (Figure S5A). Consequently, the secondary structure of this RNA fragment could be beneficial to both the stability and translation of its downstream mRNA. Similarly, the activity of the fragment −251∼−31 was higher than that of the fragment −251∼−98 (Figure 5C), and the fragment −98∼−31 did not share promoter activity (data not shown). A perfect stem-loop structure was also predicted in the secondary structure of the RNA transcript from −98∼−31 (Figure S5B). Accordingly, this stem-loop structure held by the fragment −98∼−31 could also be beneficial to mRNA stability.

### Application of Complex P*hj3*

#### Application of the 5′-UTR DNA rigion from complex P*hj3*

**Figure 6:**
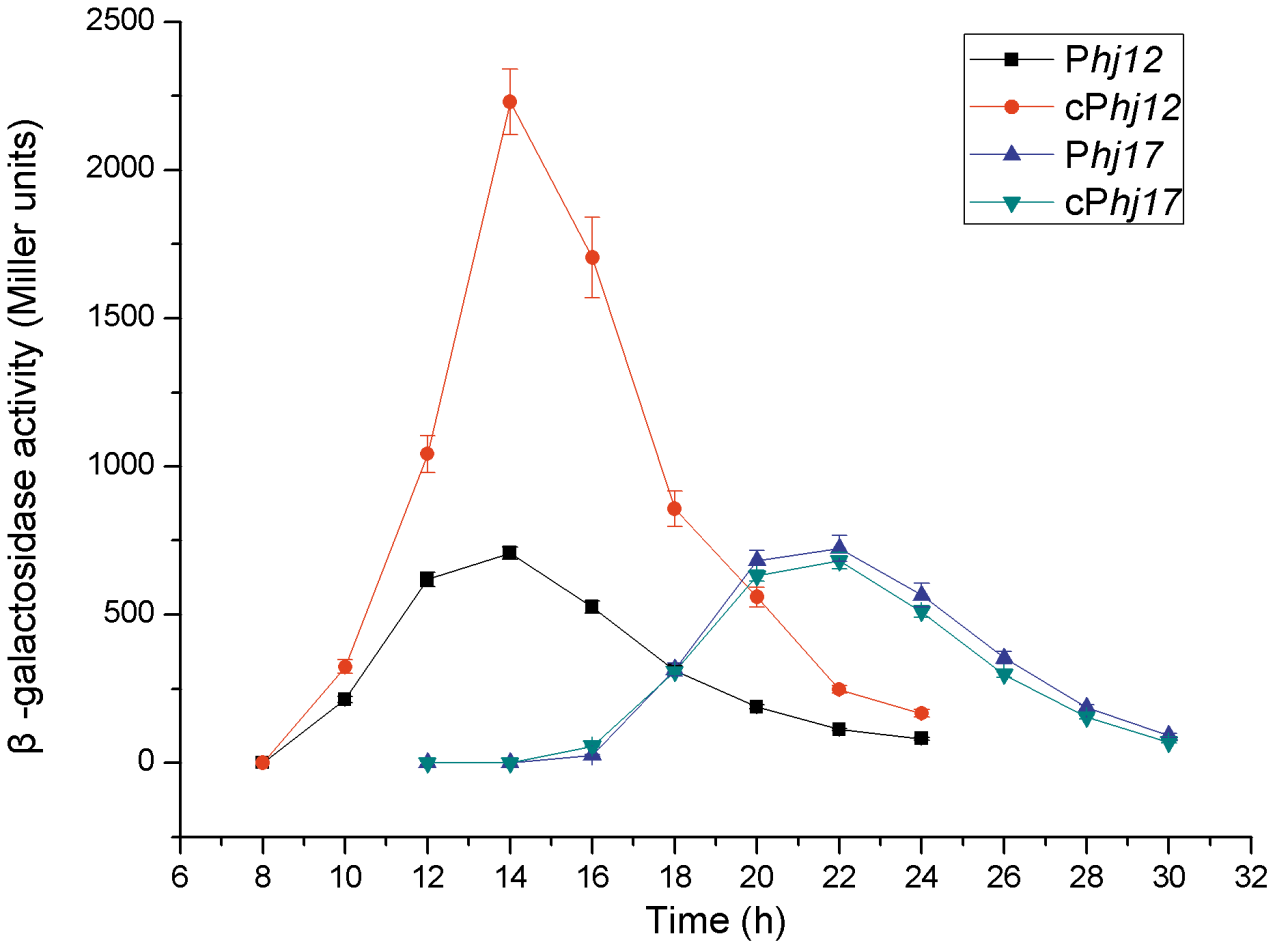
Activity analyses of the chimera complexes cP*hj12* and cP*hj17*. The 5′-UTR DNA fragment +1∼+118 of complex P*hj3* was fused at the 3′-end of the promoters of complexes P*hj12* and P*hj17* (deleting their own 5′-UTR DNA region) to construct the chimeric complexes cP*hj12* and cP*hj17*, respectively. The chimeric complexes were separately fused with the gene *lacZ* and their activities were monitored by detecting the β-galactosidase specific activities. Data are averages of three independent experiments (error bars are SEM from mean values).

Because the 5′-UTR +1∼+118 transcripted from complex P*hj3* would have some important roles in both the stability and translational facilitation of its downstream mRNA, we wondered whether or not this 5′-UTR could improve the gene expression levels directed by other weak promoters. Therefore, the DNA fragment +1∼+118 of complex P*hj3* was fused to the 3′-ends of the promoters of P*hj12* and P*hj17* complexes (deleting their own 5′-UTR DNA rigions) to construct the chimeric complexes cP*hj12* and cP*hj17*, respectively. As expected, the activity of the chimeric complex cP*hj13* increased two to three times compared to the original P*hj12* (Figure 6). Furthermore, the chimeric complex cP*hj12* exhibited the same transcriptional feature of the original complex: initial detection starting at 10 h and reaching the maximum induction at 14 h of growth (Figure 6). Unexpectedly, the activity of the chimeric complex cP*hj17* remained almost unchanged (Figure 6). These results imply that there exists some degree of context dependency between the 5′-UTR DNA region and its upstream promoter sequences.

#### Overexpression of heterologous proteins directed by complex P*hj3*

**Figure 7:**
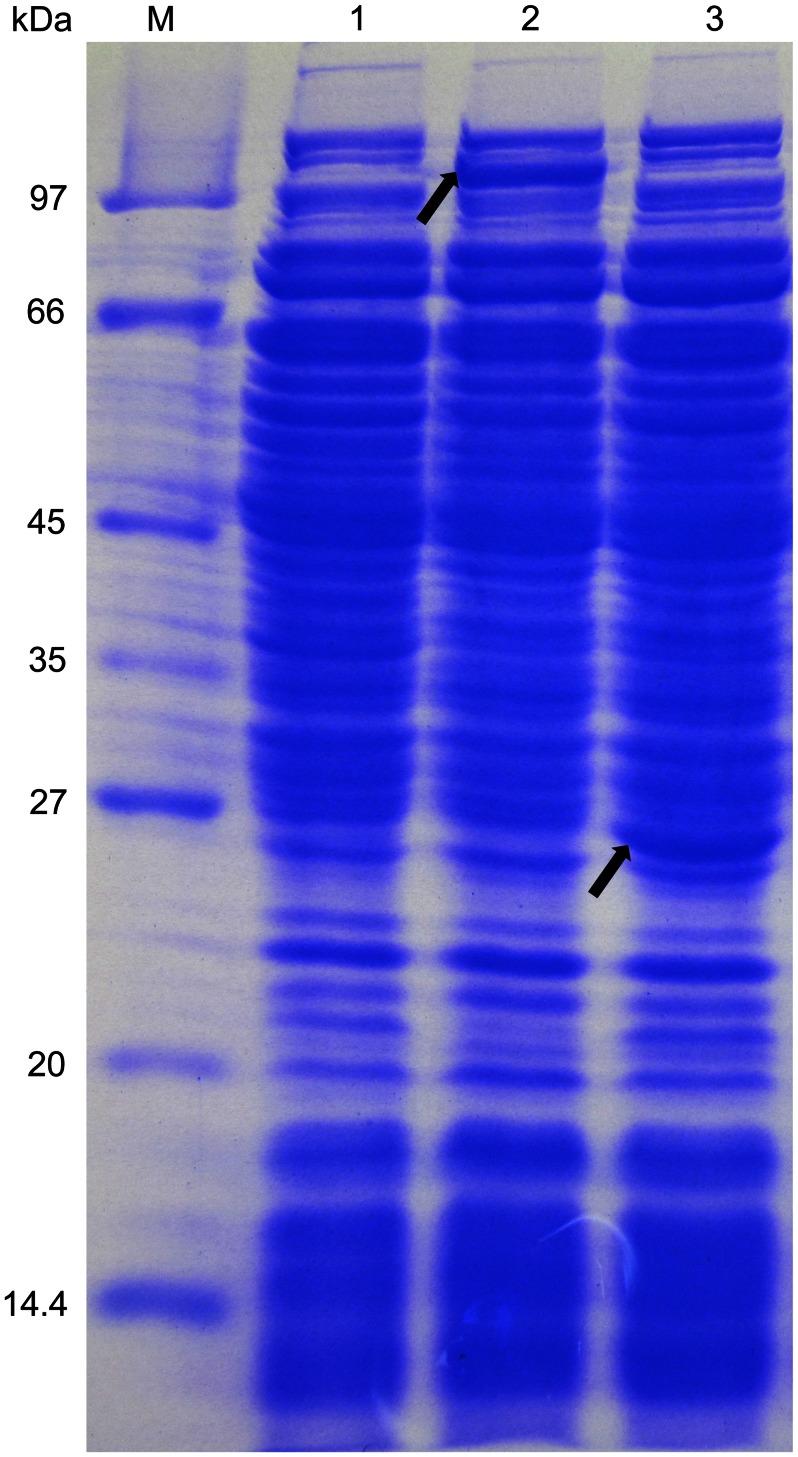
SDS-PAGE analyses of the β-galactosidase and turbo-RFP. M: Marker; 1, the strain BMB171 containing pHT1K; 2, the strain BMB171 containing pHT1K-P*hj3*-*lacZ*; 3, the strain BMB171 containing pHT1K-P*hj3*-*turbo*-*rfp*. The recombinant BMB171 strains were grown in LB medium with 25 µg/mL erythromycin at 28°C for 22 h. The cultures were harvested by centrifugation and the crude proteins were extracted by boiling. The protein bands of β-galactosidase and turbo-RFP are marked by the arrows in the lanes 2 and 3, respectively.

To evaluate whether complex P*hj3* could perform overexpression of heterologous proteins, different expression plasmids were constructed and transformed into the strain BMB171. Our results showed that the genes *lacZ* and *turbo-rfp* were successfully overexpressed with the active β-galactosidase (Figure 2A and Figure 7) and turbo-RFP (Figure 7 and Figure S6). In addition, complex P*hj3* was successfully used to overexpress some endogenous genes from *B. thuringiensis*, including the genes that encode the response regulators of the two-component system as well as the diguanylate cyclases and phosphodiesterase of the c-di-GMP-mediated signal transduction system (unpublished data).

## Discussion

### High-throughput Identification of Active Promoter Candidates

According to *in silico* prediction of the genome-wide operons (http://csbl1.bmb.uga.edu/OperonDB/), there are 4063 transcriptional units (TUs) in the genome of *B*. *thurigiensis* CT-43. In fact, only a part of TUs were transcribed under our experimental condition, simultaneously some transcribed mRNA were removed during the experimental process of RNA-seq, so the transcriptional percentages of the TUs encoded by the CT-43 chromosome were only 40.9%, 43.1%, 53.2%, and 17.7% for the four growth phases, respectively [27]. More importantly, TSSs were unable to be unambiguously determined owing to the relatively low signal-to-noise ratio for many genes with low transcriptional level. Based on the transcriptome data of *B*. *thuringiensis* CT-43 at four different growth phases, we manually determined the genome-wide TSSs and successfully identified 1203 active promoter candidates. Furthermore, we revealed their different temporal characteristics through the analyses of transcription strength at various phases coupled with secure binding sites for specific σ-factors. Therefore, from a methodological point of view, the strategy has obvious superiority on high-throughput identification of the temporally-activated promoters.

The putative binding sites for 11 different σ-factors were found in 723 active promoter candidates. The most frequently found σ-factor binding sites were those for the housekeeping σ-factor, SigA (17.4%) as well as the sporulation-specific σ-factors, SigH (15.8%), SigE (10.7%), SigG (9.3%), SigF (8.7%), and SigK (6.0%) (Table S3). These results reflect that a large number of genes are controlled by the spatially and temporally activated sporulation-specific σ-factors during sporulation [21]. In addition, these characteristics could have specific applications for gene expression research.

The 5′-UTRs of bacterial mRNAs are also known to play important regulatory roles in gene expression through extremely diverse mechanisms [9]–[17]. Among the 1203 TUs that the TSSs were mapped in this study, the length of most (52%) 5′-UTRs varied between 10 and 50 nucleotides (Table S3). In *Helicobacter pylori*, approximately 50% of the 5′-UTRs are 20–40 nucleotides in length [8], and the most frequent 5′-UTR length is also between 20 to 40 nucleotides in *E. coli* [6], whereas only 26.6% of the 5′-UTRs were 20–40 nucleotides in length in our data. In addition, very few 5′-UTRs are shorter than 20 nucleotides in *E. coli* [6], but 16.3% of the 5′-UTRs were shorter than 10 nucleotides in this study. These results might reflect the significant difference of 5′-UTR length in different species.

### The Superiority of BMB171 as a Host Strain

The wild-type strain CT-43 holds ten plasmids with different sizes and its efficiency of transformation by electroporation is very low (10^3^) [25], [37], therefore making genetic operation difficult. Fortunately, the acrystalliferous mutant BMB171 of *B. thuringiensis* YBT-1463 [32] possesses very high efficiency of electroporation transformation (10^10^) [37] and has been used as a host strain of genetic studies for a long time. Furthermore, the complete genomes of CT-43 and BMB171 have been sequenced by our laboratory [25], [32], and excellent collinearity exists in the two genomes (Figure S7). Consequently, all recombinant plasmids for the analyses of promoter-5′-UTR DNA region complex activities were transformed into strain BMB171.

### Temporal Activation of the Promoter-5′-UTR DNA Region Complex

Our results explicitly reveal the directly corresponding relationship between the σ-factor-recognized consensus sequence and the complex activity feature. The great majority of the complexes acting throughout the life cycle possess the σ^A^-like consensus sequences; some complexes that specifically exert their functions in early-stationary phase and mid-stationary phase have the σ^E^-like and σ^K^-like consensus sequences (Table S5), respectively. Our results indicate that 1) the fragment −113∼−31 of complex P*hj3* containing the σ^F^-like consensus sequence reached the maximum induction at 10 h (Figure 5C); 2) the promoters of complexes P*hj10*, P*hj11*, P*hj12*, and P*hj14* share the σ^E^-like consensus sequences, and therefore they all reached the maximum activities at approximately 14 h of growth (Figure 3); 3) the maximum activity of the σ^G^-dependent complex P*hj15* appeared at 16 h of growth (Figure 3); and 4) the promoters of complexes P*hj17*, P*hj18*, P*hj19* and P*hj20* have the σ^K^-like consensus sequence, and thus they all began induction after approximately 16 h of growth and reached maximum activity at 22 h (Figure 4). These results are consistent with the temporally-activated processes of the sporulation-specific σ-factors SigF, SigE, SigG, and SigK in *B. thuringiensis* [21]–[23].

Regarding the complexes acting throughout the life cycle, P*hj3* was confirmed to have the strongest activity, followed by P*hj2* (Figure 2). The genes directed by complexes P*hj3* and P*hj2* in CT-43 encode the 50S ribosomal protein L21 RplU and the cold shock protein CspB2, respectively. It has been shown that bacterial cold shock proteins can function as mRNA chaperones and transcription antiterminators in response to the temperature downshift and other various stresses [38], [39]. Moreover, both RplU and CspB2 have been confirmed to be highly abundant proteins by our proteomics analysis using isobaric tags for relative and absolute quantitation (iTRAQ) technique (data not shown). Consequently, complexes P*hj3* and P*hj2* as well as their cognate genes *rplU* and *cspB*2 could play important regulatory roles in the process of translation and transcription.

### The Application Prospect of the Promoter-5′-UTR DNA Region Complexes

In this study, we identified some important promoter-5′-UTR DNA region complexes that could exert their functions at specific growth phases with different activity levels. Therefore, these complexes would have different applications. For example, they could be used to investigate the gene functions in *B. thuringiensis* and other species of the *B. cereus* group. In this respect, the complexes specifically activated at certain growth phases have great significance, because the accuracy of temporal auto-induction could be superior to artificial induction. Thus, these types of complexes could be used to analyze the functions of a gene at different growth phases more precisely. In addition, the complexes with different activity levels could be used to reveal the effects of a gene on bacterial physiologic processes under its different expression levels.

More importantly, some bacilli (such as *B*. *brevis*, *B*. *megaterium* and *B*. *subtilis*) have been the most popular organism for heterologous protein production [40]. Bacilli have some general advantages, such as the lack of the endotoxin lipopolysaccharide, which is a pyrogenic factor in humans or other mammals, and the strong secretion capacity for the production of secreted enzymes [40], [41]. However, these strains also have some disadvantages leading to the poor stability of protein production, mainly because of two reasons: the very high protease activity and poor plasmid stability [42]. In contrast, some *B. thuringiensis* strains exhibit excellent plasmid compatibility and stability. For example, the strain CT-43 and YBT-1520 hold 10 and 11 plasmids with different size, respectively [25], [43]. Furthermore, the ICP proteins can be assembled into parasporal crystals, protecting the proteins from the proteolytic degradation. Meanwhile, the acrystalliferous mutant BMB171 of *B. thuringiensis* possesses some unique features, including high efficiency of electroporation transformation (10^10^) [37], excellent plasmid compatibility and stability, and clear genetic background [32]. Consequently, the strain BMB171 could be reformed to be a novel host strain for the expression of heterologous proteins.

An appropriate promoter-5′-UTR DNA region complex within a plasmid is very important regular element for the optimal overexpression of proteins. Our results confirmed that complex P*hj3* could successfully promote expression of the active β-galactosidase and turbo-RFP with sufficiently high levels (Figure 2A, Figure 7 and Figure S6). Moreover, the high expression level of heterologous proteins did not significantly affect the growth features of the recombinant BMB171 strains (data not shown). Thus, P*hj3* would be a proper promoter-5′-UTR DNA region complex for the overexpression of proteins in the strain BMB171.

In conclusion, the results of this study provide a substantial contribution to molecular biology research and biotechnological applications of *B. thuringiensis*, and our work has made the first step in developing a novel protein expression system in this regard.

## Supporting Information

Figure S1 **Flow chart of the construction of the translational fusion plasmid pHT1K-P** ***hj1*** **-** ***lacZ*** **.**
(TIF)

Figure S2 **Flow chart of the construction of the expression plasmid pHT1K-P** ***hj3*** **-** ***turbo-rfp*** **.**
(TIF)

Figure S3 **Visualization of TSS mapping for P** ***hj1*** **-P** ***hj20*** **.** The number of unambiguously mapped reads per nucleotide was calculated and visualized by R and Origin version 8.0. The black, red, blue and dark cyan columns represent the mapped reads per nucleotide at 7 h, 9 h, 13 h and 22 h, respectively. The green and purple arrows represent the coordinates of a complex and the first downstream ORF, respectively.
(PDF)

Figure S4 **Distribution of individual 5′-UTR length based on 1203 TSSs of mRNAs.**
(TIF)

Figure S5 The predicted RNA secondary structures of the fragments +1∼ +118 (A) and −106∼ −31 (B) transcripted from P*hj3*. RNA secondary structures were predicted by Mfold (version 2.3) at 28°C based on global minimum free energy principle with no constrains [36], and visualized by VARNA.
(TIF)

Figure S6 **Activity analysis of turbo-RFP.** The strain BMB171 containing pHT1K (left) and pHT1K-P*hj3*-*turbo*-*rfp* (right) were inoculated on the same LB plate analysis detected in plate with 25 µg/mL erythromycin. The magenta bacterial colonies demonstrated that these bacterial cells produced the active turbo-RFP.
(TIF)

Figure S7 **Nucleotide alignment obtained by the program MUMmer.** Whole-genome sequence comparison was performed at the nucleotide level using the program MUMmer (http://mummer.sourceforge.net/) with default values, which relies on exact matches of at least 20 base pairs. Each dot in the figure is one such match. The red lines on the two main diagonals result from the high density of points with sequence identity along chromosome of the two *Bacillus thuringiensis* strains CT-43 and BMB171. The scattered points outside the main diagonals represent other short regions of sequence identity.
(TIF)

Table S1 Bacterial strains and plasmids used in this study.
(DOC)

Table S2 Primers used in this study.
(DOC)

Table S3 Genome-wide TSS mapping and promoter identification.
(XLS)

Table S4 Highly active complex candidates selected in this study.
(DOC)

Table S5 Consensus sequences recognized by the σ-factor in the complex candidates.
(DOC)
