# Supplementary figures and images for: High-Throughput Identification of Promoters and Screening of Highly Active Promoter-5′-UTR DNA Region with Different Characteristics from Bacillus thuringiensis
Source: PLoS One. 2013 May 10;8(5):e62960. doi: 10.1371/journal.pone.0062960 (PMC3651082; doi:10.1371/journal.pone.0062960)

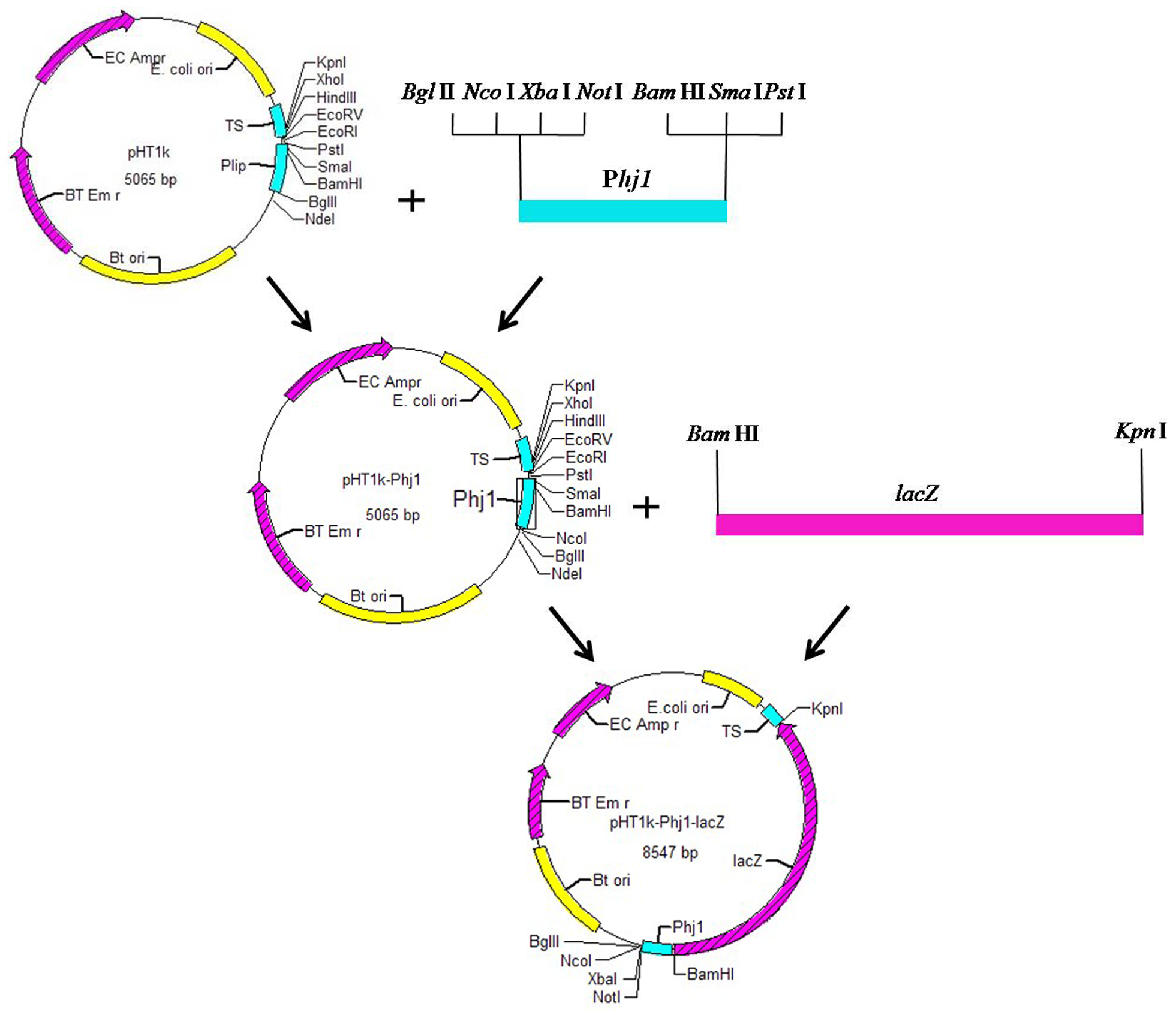

Supplement: Figure S1 — Flow chart of the construction of the translational fusion plasmid pHT1K-P hj1 - lacZ . (TIF) [file pone.0062960.s001.tif]

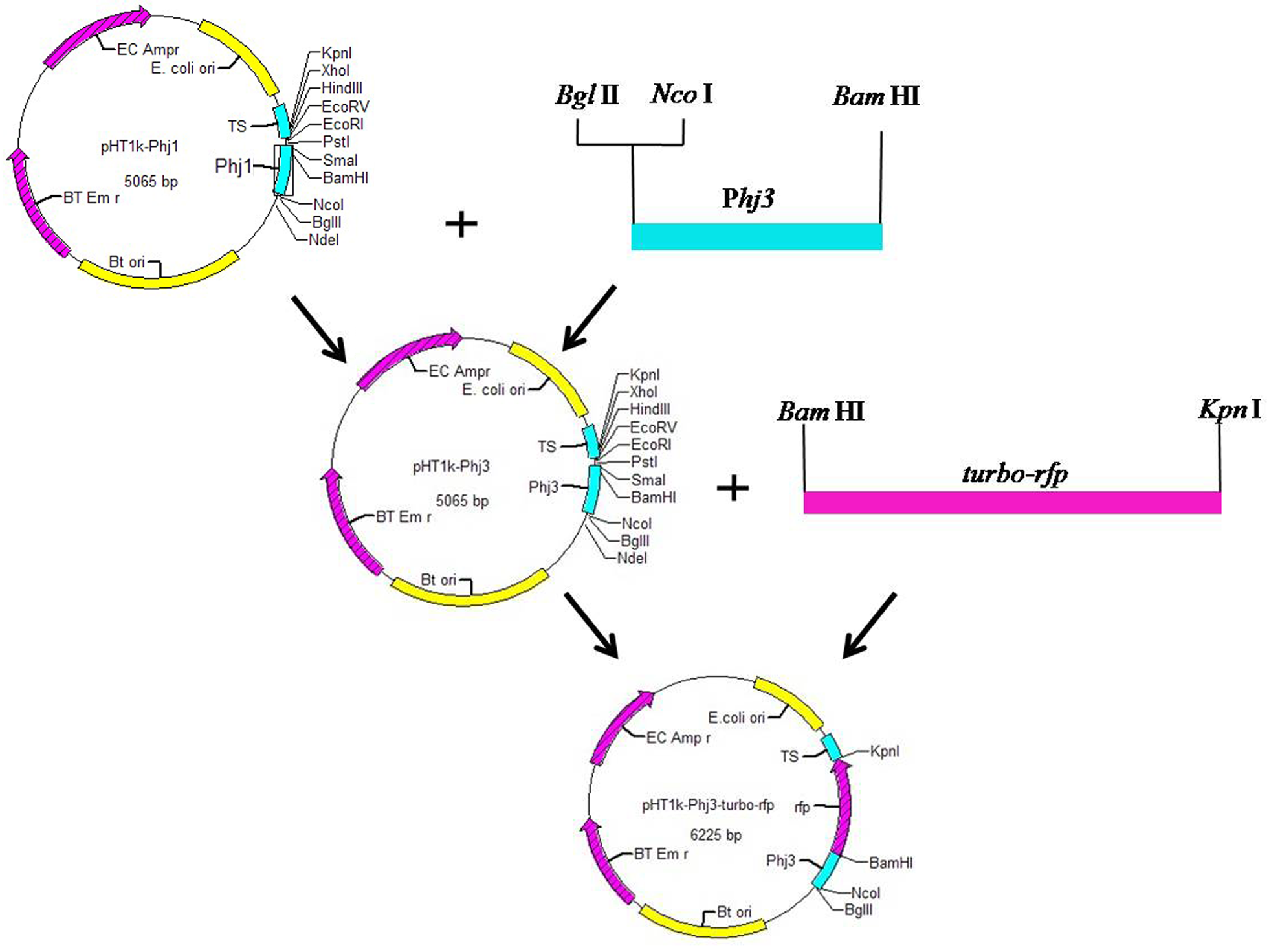

Supplement: Figure S2 — Flow chart of the construction of the expression plasmid pHT1K-P hj3 - turbo-rfp . (TIF) [file pone.0062960.s002.tif]

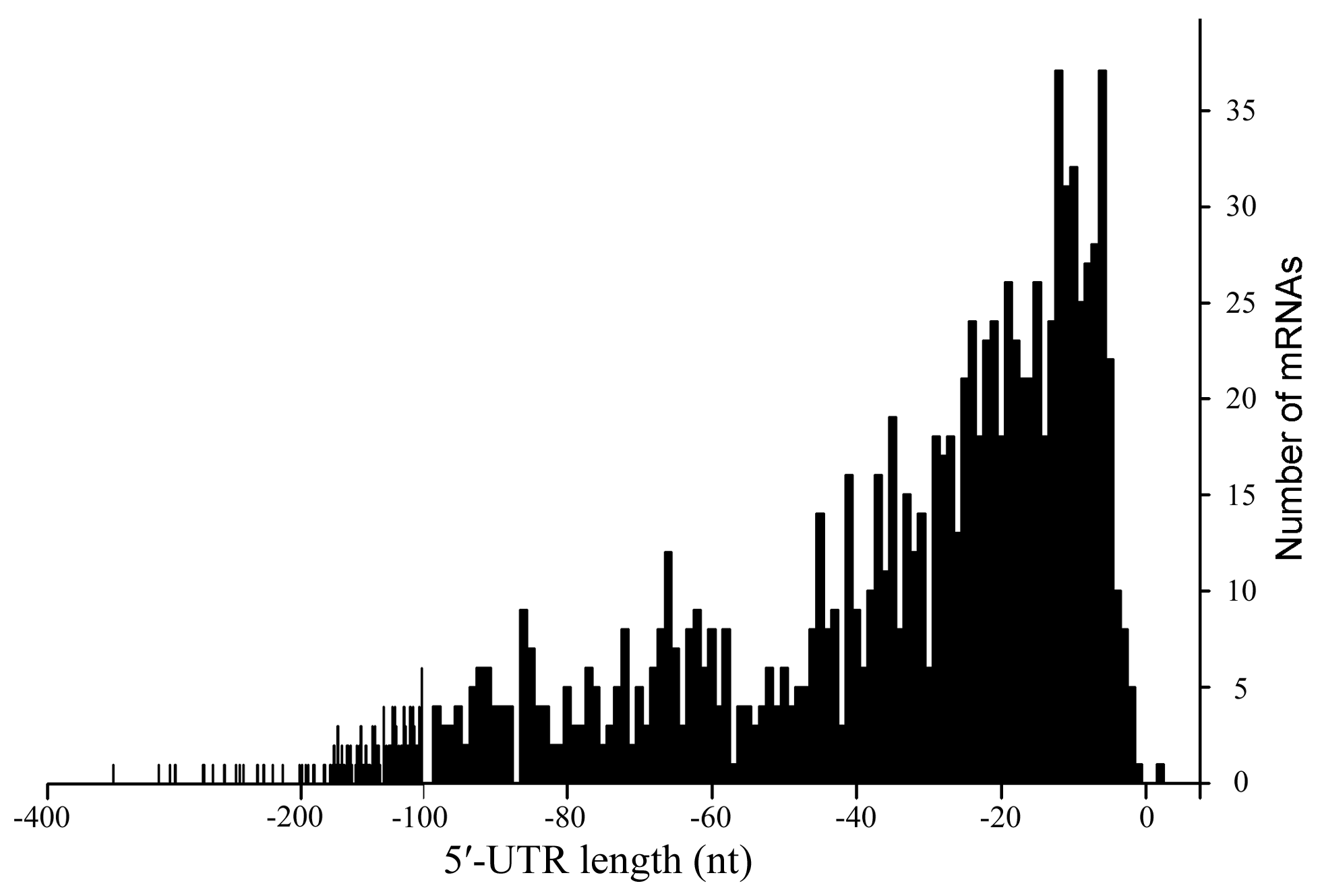

Supplement: Figure S4 — Distribution of individual 5′-UTR length based on 1203 TSSs of mRNAs. (TIF) [file pone.0062960.s004.tif]

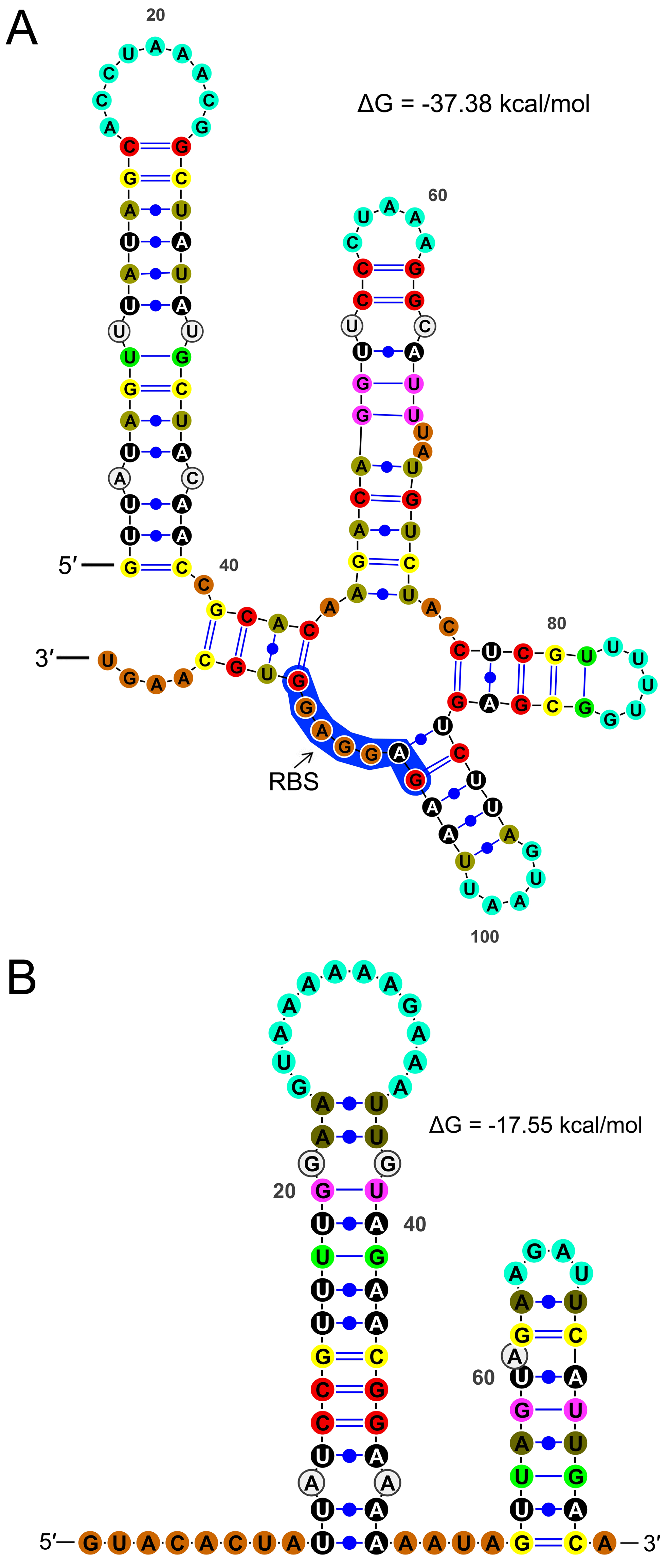

Supplement: Figure S5 — The predicted RNA secondary structures of the fragments +1∼ +118 (A) and −106∼ −31 (B) transcripted from Phj3. RNA secondary structures were predicted by Mfold (version 2.3) at 28°C based on global minimum free energy principle with no constrains [36], and visualized by VARNA. (TIF) [file pone.0062960.s005.tif]

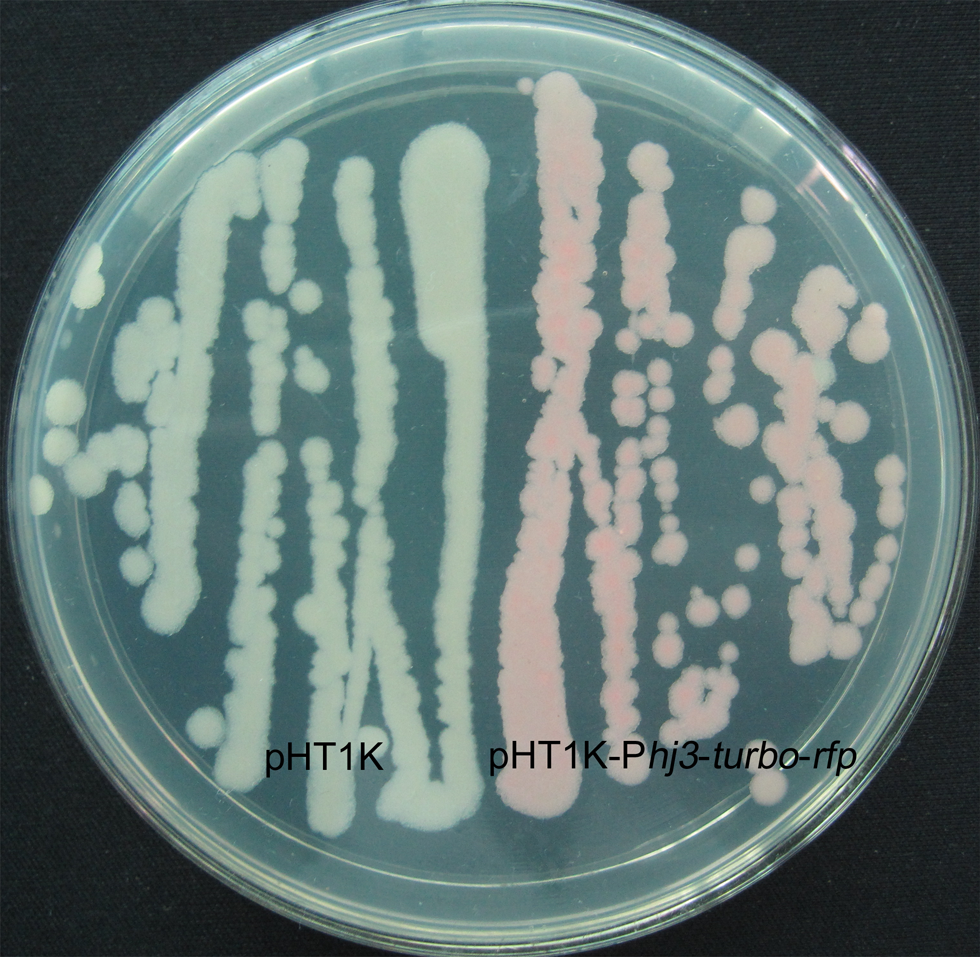

Supplement: Figure S6 — Activity analysis of turbo-RFP. The strain BMB171 containing pHT1K (left) and pHT1K-Phj3-turbo-rfp (right) were inoculated on the same LB plate analysis detected in plate with 25 µg/mL erythromycin. The magenta bacterial colonies demonstrated that these bacterial cells produced the active turbo-RFP. (TIF) [file pone.0062960.s006.tif]

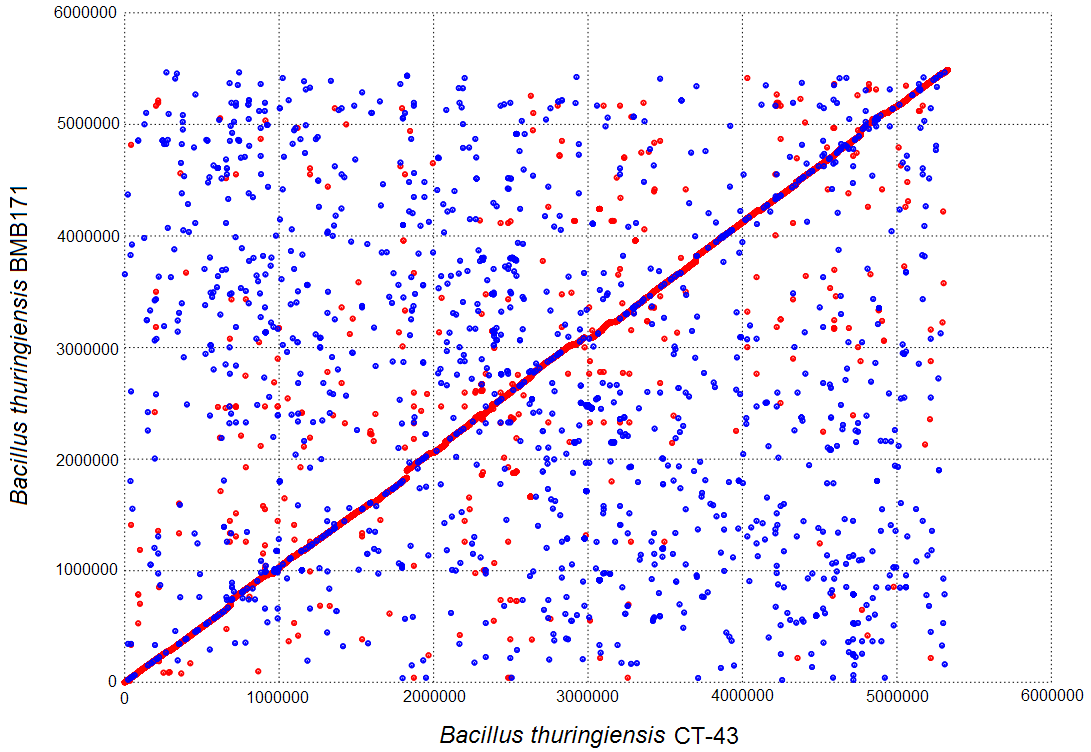

Supplement: Figure S7 — Nucleotide alignment obtained by the program MUMmer. Whole-genome sequence comparison was performed at the nucleotide level using the program MUMmer (http://mummer.sourceforge.net/) with default values, which relies on exact matches of at least 20 base pairs. Each dot in the figure is one such match. The red lines on the two main diagonals result from the high density of points with sequence identity along chromosome of the two Bacillus thuringiensis strains CT-43 and BMB171. The scattered points outside the main diagonals represent other short regions of sequence identity. (TIF) [file pone.0062960.s007.tif]
