# Supplementary material for: High-Throughput Identification of Promoters and Screening of Highly Active Promoter-5′-UTR DNA Region with Different Characteristics from Bacillus thuringiensis
Source: PLoS One. 2013 May 10;8(5):e62960. doi: 10.1371/journal.pone.0062960 (PMC3651082; doi:10.1371/journal.pone.0062960)
Supplement: Figure S3 — Visualization of TSS mapping for P hj1 -P hj20 . The number of unambiguously mapped reads per nucleotide was calculated and visualized by R and Origin version 8.0. The black, red, blue and dark cyan columns represent the mapped reads per nucleotide at 7 h, 9 h, 13 h and 22 h, respectively. The green and purple arrows represent the coordinates of a complex and the first downstream ORF, respectively. (PDF) [file pone.0062960.s003.pdf]

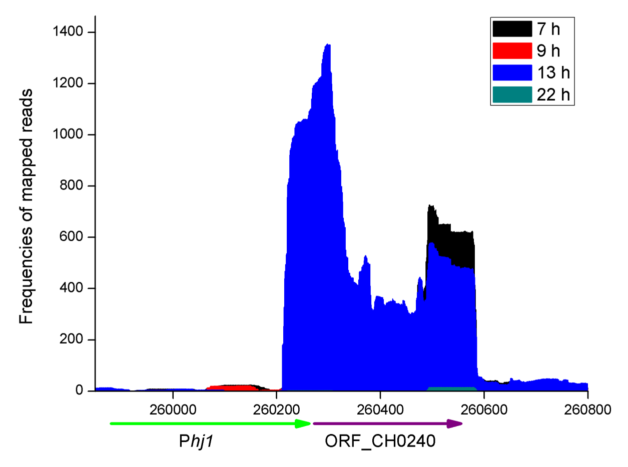

7 h
9 h
13 h
22 h
Frequencies of mapped reads
0
200
400
600
800
1000
1200
1400
260000
260200
260400
260600
260800
Phj1
ORF_CH0240

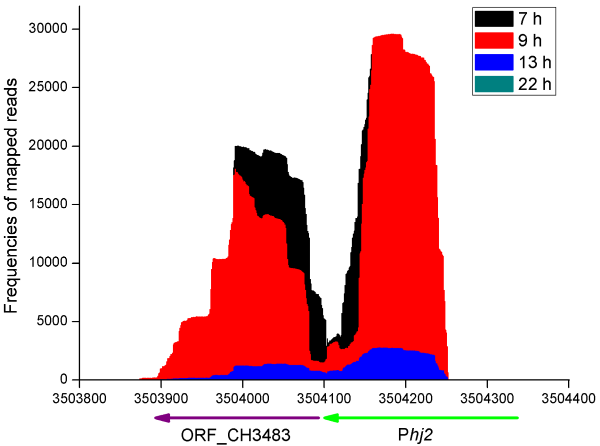

7 h
9 h
13 h
22 h
Frequencies of mapped reads
0
5000
10000
15000
20000
25000
30000
3503800
3503900
3504000
3504100
3504200
3504300
3504400
ORF_CH3483
Phj2

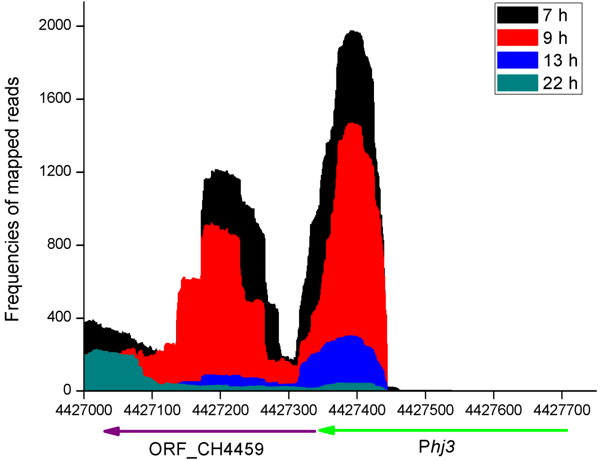

7 h
9 h
13 h
22 h
Frequencies of mapped reads
0
400
800
1200
1600
2000
4427000
4427100
4427200
4427300
4427400
4427500
4427600
4427700
ORF_CH4459
P*hj3*

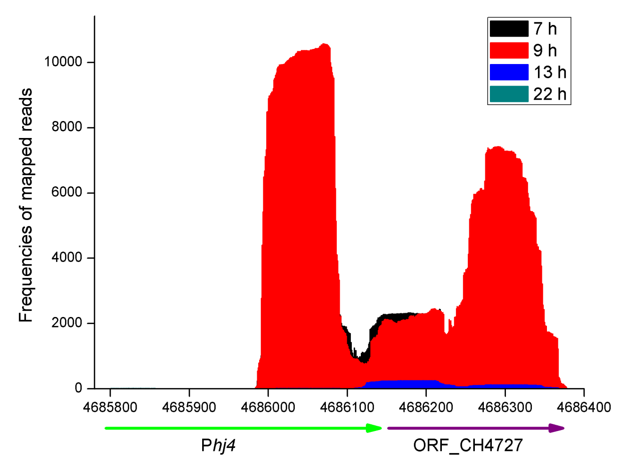

7 h
9 h
13 h
22 h
Frequencies of mapped reads
0
2000
4000
6000
8000
10000
4685800
4685900
4686000
4686100
4686200
4686300
4686400
P*hj4*
ORF_CH4727

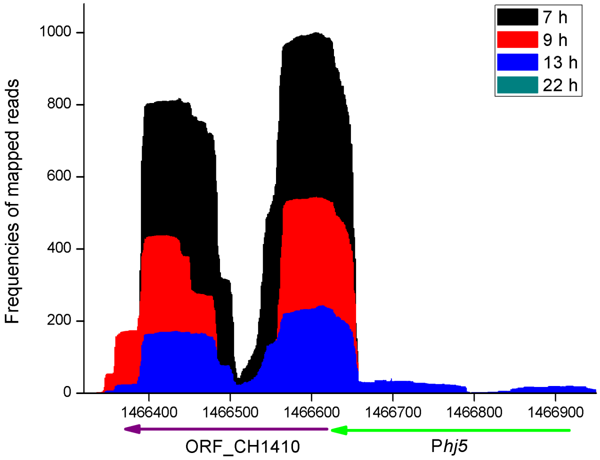

Frequencies of mapped reads
0
200
400
600
800
1000
1466400
1466500
1466600
1466700
1466800
1466900
7 h
9 h
13 h
22 h
ORF_CH1410
P*hj5*

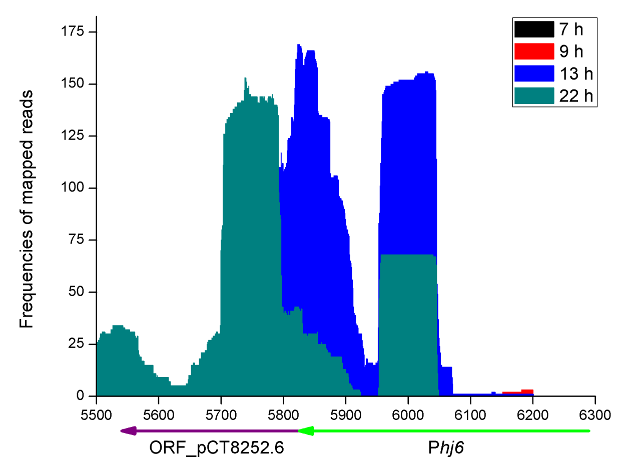
7 h
9 h
13 h
22 h
Frequencies of mapped reads
0
25
50
75
100
125
150
175
5500
5600
5700
5800
5900
6000
6100
6200
6300
ORF_pCT8252.6
P*hj6*

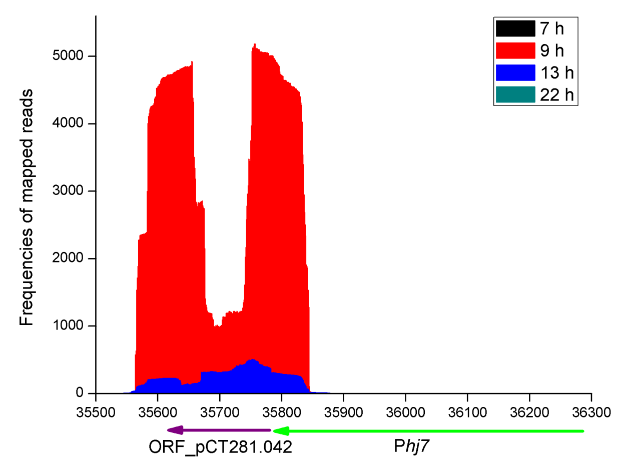

7 h
9 h
13 h
22 h
Frequencies of mapped reads
0
1000
2000
3000
4000
5000
35500
35600
35700
35800
35900
36000
36100
36200
36300
ORF_pCT281.042
Phj7

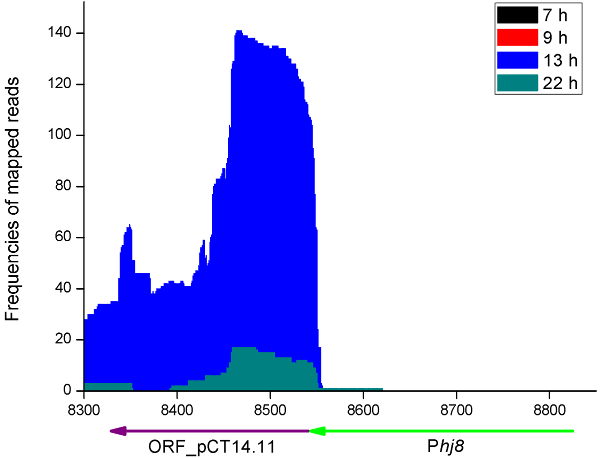

Frequencies of mapped reads
0
20
40
60
80
100
120
140
8300
8400
8500
8600
8700
8800
7 h
9 h
13 h
22 h
ORF_pCT14.11
P*hj8*

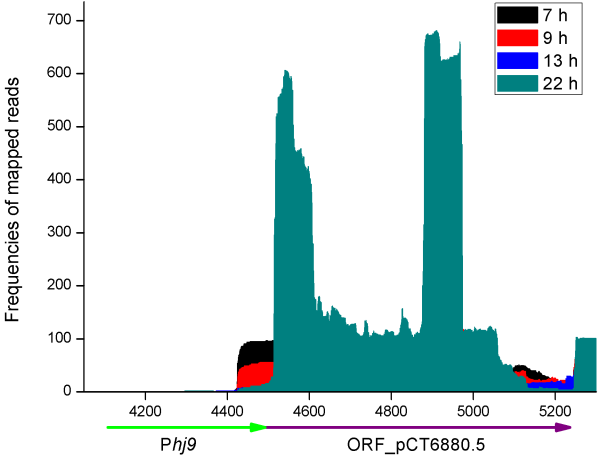

Frequencies of mapped reads
0
100
200
300
400
500
600
700
4200
4400
4600
4800
5000
5200
7 h
9 h
13 h
22 h
P*hj9*
ORF_pCT6880.5

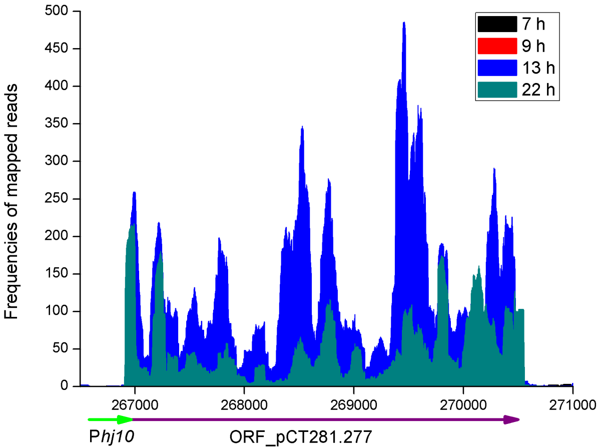

7 h
9 h
13 h
22 h
Frequencies of mapped reads
0
50
100
150
200
250
300
350
400
450
500
267000
268000
269000
270000
271000
P*hj10*
ORF_pCT281.277

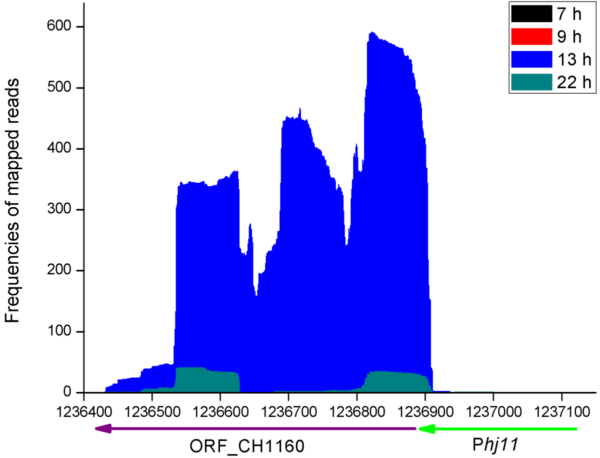

Frequencies of mapped reads
600
500
400
300
200
100
0
1236400
1236500
1236600
1236700
1236800
1236900
1237000
1237100
7 h
9 h
13 h
22 h
ORF_CH1160
P*hj11*

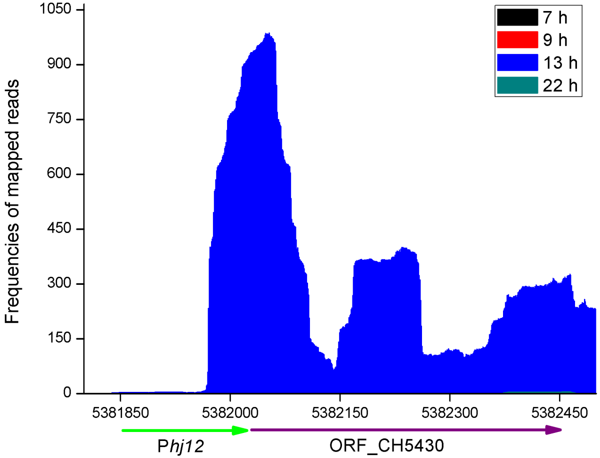

Frequencies of mapped reads
1050
900
750
600
450
300
150
0
5381850
5382000
5382150
5382300
5382450
7 h
9 h
13 h
22 h
Phj12
ORF_CH5430

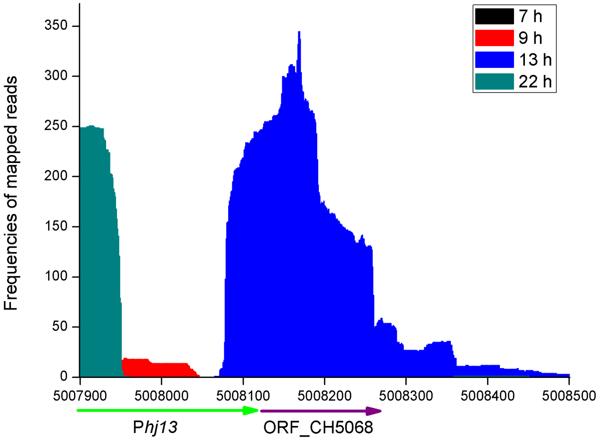

7 h
9 h
13 h
22 h
Frequencies of mapped reads
0
50
100
150
200
250
300
350
5007900
5008000
5008100
5008200
5008300
5008400
5008500
Phj13
ORF_CH5068

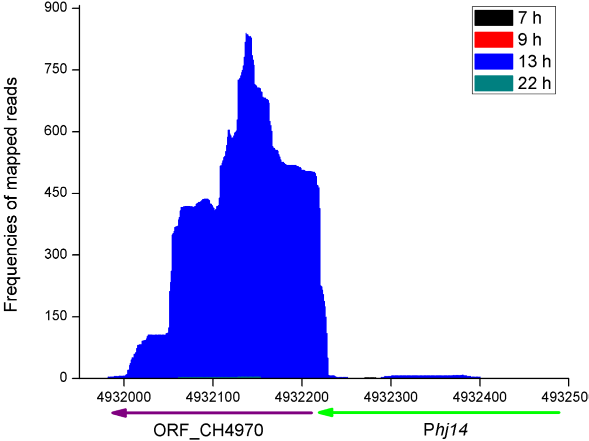

900
750
600
450
300
150
0
Frequencies of mapped reads
7 h
9 h
13 h
22 h
4932000
4932100
4932200
4932300
4932400
4932500
ORF_CH4970
Phj14

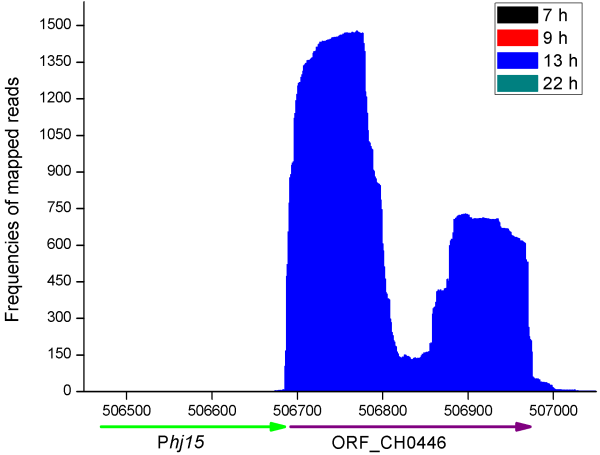

7 h
9 h
13 h
22 h
Frequencies of mapped reads
0
150
300
450
600
750
900
1050
1200
1350
1500
506500
506600
506700
506800
506900
507000
Phj15
ORF_CH0446

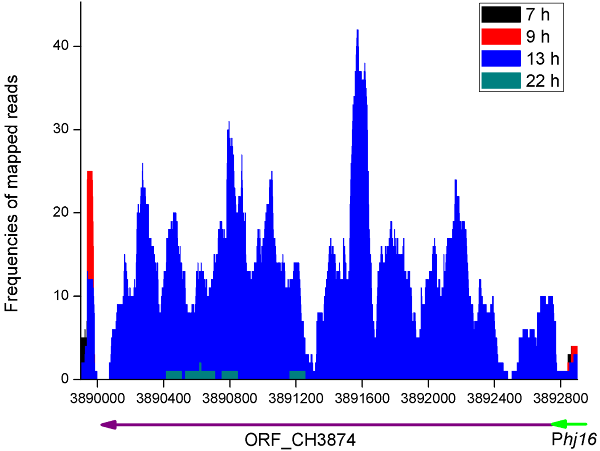

7 h
9 h
13 h
22 h
Frequencies of mapped reads
0
10
20
30
40
3890000
3890400
3890800
3891200
3891600
3892000
3892400
3892800
ORF_CH3874
Phj16

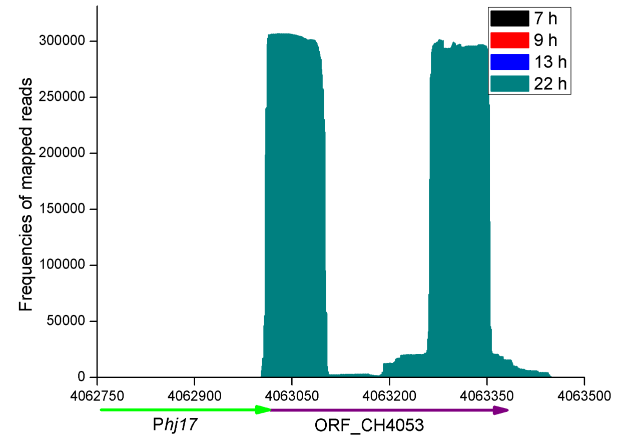

7 h
9 h
13 h
22 h
Frequencies of mapped reads
300000
250000
200000
150000
100000
50000
0
4062750
4062900
4063050
4063200
4063350
4063500
Phj17
ORF_CH4053

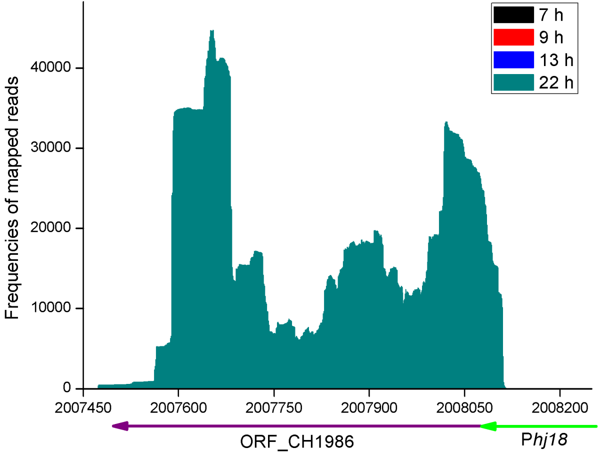
7 h
9 h
13 h
22 h
Frequencies of mapped reads
0
10000
20000
30000
40000
2007450
2007600
2007750
2007900
2008050
2008200
ORF_CH1986
Phj18

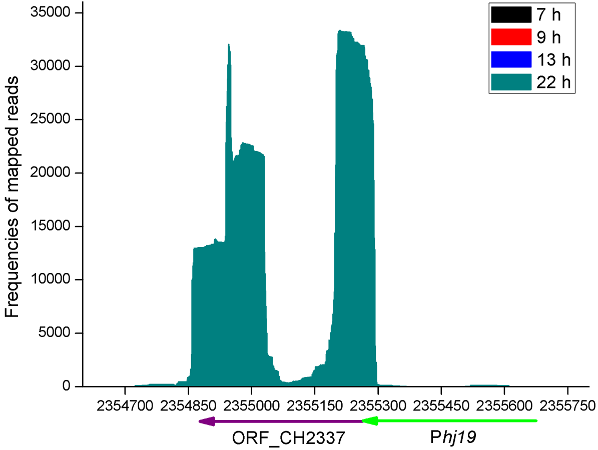

Frequencies of mapped reads
35000
30000
25000
20000
15000
10000
5000
0
2354700
2354850
2355000
2355150
2355300
2355450
2355600
2355750
7 h
9 h
13 h
22 h
ORF_CH2337
Phj19

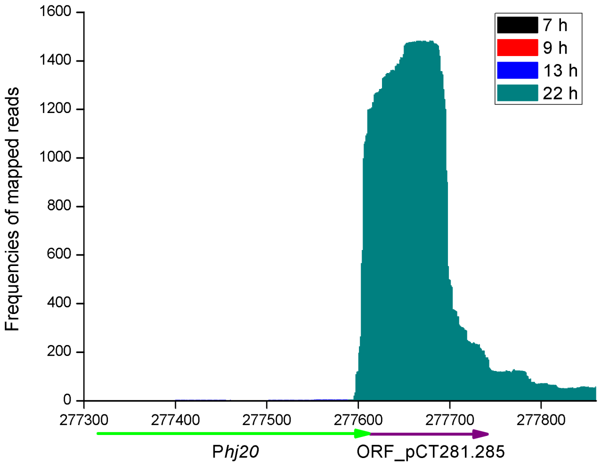
1600
1400
1200
1000
800
600
400
200
0
Frequencies of mapped reads
7 h
9 h
13 h
22 h
277300
277400
277500
277600
277700
277800
P*hj20*
ORF_pCT281.285
