# Supplementary material for: High-Throughput Identification of Promoters and Screening of Highly Active Promoter-5′-UTR DNA Region with Different Characteristics from Bacillus thuringiensis
Source: PLoS One. 2013 May 10;8(5):e62960. doi: 10.1371/journal.pone.0062960 (PMC3651082; doi:10.1371/journal.pone.0062960)
Supplement: Table S1 — Bacterial strains and plasmids used in this study. (DOC) [file pone.0062960.s008.doc]

**Table S1 Bacterial strains and plasmids used in this study**

| **Materials** | **Characteristics** | **Source** |
| --- | --- | --- |
| **Strains** | | |
| *E. coli* DH5α | RecA1 endA1 gyrA96 thi hsdR17(r_k_^-^ m_k_^+^) relA1 supE44 Φ80△lacZ△M15△(lacZYA-argF)U169 | Stored by our Lab |
| BMB171 | *B. thuringiensis* strain BMB171; an acrystalliferous mutant strain; high transformation frequency | Isolated by our Lab |
| CT-43 | *B. thuringiensis* strain CT-43 carrying the *cry*1Aa3, *cry*1Ba1, *cry*1Ia14, *cry*2Aa9, and *cry*2Ab1 genes | Isolated by our Lab |
| **Plasmids** | | |
| pHT1K (pHT1000) | *B. thuringiensis*-*E. coli* shuttle plasmid; Amp^r^ Erm^r^ | [30] |
| pRP1028 | Amp^r^ Erm^r^; containing *turbo-rfp* gene | a gift from Scott Stibitz, Center for Biologics Evaluation and Research, Food and Drug Administration, Bethesda, Maryland, USA |
| pHT304-18Z | Amp^r^ Erm^r^; *E. coli*-*B. thuringiensis* shuttle vector harboring the promoterless *lacZ* gene | [[31]](#_ENREF_1) |
| **Translational fusion plasmids** | | |
| pHT1K-P*hj1* | pHT1K carrying P*hj1* | This study |
| pHT1K-P*hj1*-*lacZ* | pHT1K carrying P*hj1* and the *lacZ* gene | This study |
| pHT1K-P*hj2*-*lacZ* | pHT1K carrying P*hj2* and the *lacZ* gene | This study |
| pHT1K-P*hj3*-*lacZ* | pHT1K carrying P*hj3* and the *lacZ* gene | This study |
| pHT1K-P*hj4*-*lacZ* | pHT1K carrying P*hj4* and the *lacZ* gene | This study |
| pHT1K-P*hj5*-*lacZ* | pHT1K carrying P*hj5* and the *lacZ* gene | This study |
| pHT1K-P*hj6*-*lacZ* | pHT1K carrying P*hj6* and the *lacZ* gene | This study |
| pHT1K-P*hj7*-*lacZ* | pHT1K carrying P*hj7* and the *lacZ* gene | This study |
| pHT1K-P*hj8*-*lacZ* | pHT1K carrying P*hj8* and the *lacZ* gene | This study |
| pHT1K-P*hj9*-*lacZ* | pHT1K carrying P*hj9* and the *lacZ* gene | This study |
| pHT1K-P*hj10*-*lacZ* | pHT1K carrying P*hj10* and the *lacZ* gene | This study |
| pHT1K-P*hj11*-*lacZ* | pHT1K carrying P*hj11* and the *lacZ* gene | This study |
| pHT1K-P*hj12*-*lacZ* | pHT1K carrying P*hj12* and the *lacZ* gene | This study |
| pHT1K-P*hj13*-*lacZ* | pHT1K carrying P*hj13* and the *lacZ* gene | This study |
| pHT1K-P*hj14*-*lacZ* | pHT1K carrying P*hj14* and the *lacZ* gene | This study |
| pHT1K-P*hj15*-*lacZ* | pHT1K carrying P*hj15* and the *lacZ* gene | This study |
| pHT1K-P*hj16*-*lacZ* | pHT1K carrying P*hj16* and the *lacZ* gene | This study |
| pHT1K-P*hj17*-*lacZ* | pHT1K carrying P*hj17* and the *lacZ* gene | This study |
| pHT1K-P*hj18*-*lacZ* | pHT1K carrying P*hj18* and the *lacZ* gene | This study |
| pHT1K-P*hj19*-*lacZ* | pHT1K carrying P*hj19* and the *lacZ* gene | This study |
| pHT1K-P*hj20*-*lacZ* | pHT1K carrying P*hj20* and the *lacZ* gene | This study |
| pHT1K-cP*hj12*-*lacZ* | pHT1K carrying promoter of P*hj12* and 5΄-UTR DNA region of P*hj3* chimeric complex and the *lacZ* gene | This study |
| pHT1K-cP*hj17* -*lacZ* | pHT1K carrying promoter of P*hj17* and 5΄-UTR DNA region of P*hj3* chimeric complex and the *lacZ* gene | This study |
| **Transcriptional fusion plasmids** | | |
| pHT1K-*lacZ*(UTR) | pHT1K carrying the *lacZ* gene with its own 5΄-UTR DNA region | This study |
| pHT1K-P*hj3*(-251~-98)-*lacZ*(UTR) | pHT1K carrying the fragment -251~-98 of P*hj3* and the *lacZ* gene with its own 5΄-UTR DNA region | This study |
| pHT1K-P*hj3*(-251~-31)-*lacZ*(UTR) | pHT1K carrying the fragment -251~-31 of P*hj3* and the *lacZ* gene with its own 5΄-UTR DNA region | This study |
| pHT1K-P*hj3*(-251~+14)-*lacZ*(UTR) | pHT1K carrying the fragment -251~+14 of P*hj3* and the *lacZ* gene with its own 5΄-UTR DNA region | This study |
| pHT1K-P*hj3*(-113~-31)-*lacZ*(UTR) | pHT1K carrying the fragment -113~-31 of P*hj3* and the *lacZ* gene with its own 5΄-UTR DNA region | This study |
| pHT1K-P*hj3*(-54~+14)-*lacZ*(UTR) | pHT1K carrying the fragment -54~+14 of P*hj3* and the *lacZ* gene with its own 5΄-UTR DNA region | This study |
| pHT1K-P*hj3*(-54~+118)-*lacZ*(UTR) | pHT1K carrying the fragment -54~+118 of P*hj3* and the *lacZ* gene with its own 5΄-UTR DNA region | This study |
| pHT1K-P*hj3*(-6~+118)-*lacZ*(UTR) | pHT1K carrying the fragment -6~+118 of P*hj3* and the *lacZ* gene with its own 5΄-UTR DNA region | This study |
| **Plasmids for expression of protein** | | |
| pHT1K-P*hj3* | pHT1K carrying P*hj3* | This study |
| pHT1K-P*hj3-turbo-rfp* | pHT1K carrying P*hj3* and the *turbo-rfp* gene | This study |
