# Supplementary material for: High-Throughput Identification of Promoters and Screening of Highly Active Promoter-5′-UTR DNA Region with Different Characteristics from Bacillus thuringiensis
Source: PLoS One. 2013 May 10;8(5):e62960. doi: 10.1371/journal.pone.0062960 (PMC3651082; doi:10.1371/journal.pone.0062960)
Supplement: Table S2 — Primers used in this study. (DOC) [file pone.0062960.s009.doc]

**Table S2 Primers used in this study**

| **Primer *^a^*** | **Sequence (5**′ **→3**′**)^b^** |
| --- | --- |
| P*hj1*-F | CATG**AGATCT**AATACGACTCACTATAGGG**CCATGGTCTAGAGCGGCCGC**AATGGTTTAACCATCCTTTC |
| P*hj1*-R | AA**CTGCAGCCCGGGGGATCC**ACCTCCTCATTTTGTTATG |
| P*hj2*-F  P*hj2*-R | CATG**CCATGG**GCAATGCAGTGGGAAC  CGC**GGATCC**AATCCTCCTACAATGTAC |
| P*hj3*-F  P*hj3*-R | CATG**CCATGG**AGAGAGAATTAATCAC  CGC**GGATCC**ACCTCCTCTTAATTAC |
| P*hj4*-F  P*hj4*-R | CATG**CCATGG**TCACGCATCCATCCTTC  CGC**GGATCC**AACTCCTCTACTAGT |
| P*hj5*-F | CATG**CCATGG**GACCGCTTCCACCTTCC |
| P*hj5*-R | CGC**GGATCC**ATTCCCTCCTGATAATATG |
| P*hj6*-F  P*hj6*-R | CATG**CCATGG**TAACAAATCCGTAACC  CGC**GGATCC**TTGGACTTCCTCCTTG |
| P*hj7*-F  P*hj7*-R | CATG**CCATGG**TTATAAACCATCAGGTC  CGC**GGATCC**TACACCTCCTTTCCCTC |
| P*hj8*-F  P*hj8*-R | CATG**CCATGG**TTTTGGTTAACTGTAG  CGC**GGATCC**AAAACAACCTCCTATC |
| P*hj9*-F  P*hj9*-R | CATG**CCATGG**AAGGATGAATGTTTAC  CGC**GGATCC**AATATTCATCCCTTC |
| P*hj10*-F  P*hj10*-R | CATG**CCATGG**CATTTACAATTCAAG  CGC**GGATCC**AAGTTACCTCCATCTC |
| P*hj11*-F  P*hj11*-R | CATG**CCATGG**TTCTCCGCAATTTTTCAG  CGC**GGATCC**CACTCCTTCTCGTGAG |
| P*hj12*-F | CATG**CCATGG**ATGATGAAAAGCTAGCTCAC |
| P*hj12*-R | CGC**GGATCC**GTCACCTCTCTTTTTG |
| P*hj13*-F  P*hj13*-R | CATG**CCATGG**CTATTCTTGAATGAATG  CGC**GGATCC**ATTCCTCCAGTTTCGTC |
| P*hj14*-F  P*hj14*-R | CATG**CCATGG**GTTAAGTTCACTTCAG  CGC**GGATCC**AGTTCCTCCCCTTATATTC |
| P*hj15*-F  P*hj15*-R | CATG**CCATGG**TCTGTTGTAATGAATG  CGC**GGATCC**CACCTCCACAATCAATAATG |
| P*hj16*-F | CATG**CCATGG**TCATACTTTGAAGGTTG |
| P*hj16*-R | CGC**GGATCC**CCTGCCATCGACCTCC |
| P*hj17*-F  P*hj17*-R | CATG**CCATGG**TGTACCACTACTCCTAG  CGC**GGATCC**AATCCTCATCACCCCC |
| P*hj18*-F  P*hj18*-R | CATG**CCATGG**TGTTTTCTCATCCCTTC  CGC**GGATCC**AGAAATCCTCCTTATC |
| P*hj19*-F  P*hj19*-R | CATG**CCATGG**CTATTTTGTTATATTC  CGC**GGATCC**TTTATATTAACCTCC |
| P*hj20*-F  P*hj20*-R | CATG**CCATGG**TTGTTTTGAACTACAGC  CGC**GGATCC**GAGAATCGCCTCCAAG |
| *lacZ*-F | CGC**GGATCC**ATGGTGGAAGTTACTGACGTAAG |
| *lacZ*-R | CGG**GGTACC**TTATTATTATTTTTGACACCAGACC |
| P*hj3*(-251~-98)-R | CGC**GGATCC**ATAGTGTACTTTTCG |
| P*hj3*(-251~-31)-R | CGC**GGATCC**TGTCAATGAATCTTC |
| P*hj3*(-251~+14)-R | CGC**GGATCC**CTATAAACTATAAC |
| P*hj3*(-54~+118)-F | CATG**CCATGG**TAGTTAGTAGAAGATTC |
| P*hj3*(-6~+118)-F | CATG**CCATGG**CTTTATGTTATAGTT |
| P*hj3*(-113~-31)-F | CATG**CCATGG**CGAAAAGTACACTATTATC |
| P*hj3*(-113~-31)-R | CGC**GGATCC**TGTCAATGAATCTTCTAC |
| P*hj3*(-54~+14)-F | CATG**CCATGG**AGTTAGTAGAAGATTCATTG |
| P*hj3*(-54~+14)-R | CGC**GGATCC**CTATAAACTATAACATAAAG |
| cP*hj12*-1 | AACTATAACATAAAGTTTGTAATACCTTTTGG |
| cP*hj12*-2 | CCAAAAGGTATTACAAACTTTATGTTATAGTT |
| cP*hj17*-1 | AACTATAACATAAAGCCTTCACACTTATAATC |
| cP*hj17*-2 | GATTATAAGTGTGAAGGCTTTATGTTATAGTT |
| *turbo-rfp*-F  *turbo-rfp*-R | CGC**GGATCC**ATGAGTGAATTGATTAAAG  CGG**GGTACC**TTATCTATGCCCTAATTTAC |

***^a^*** Both the forward (F) and reverse (R) primers are listed in the 5' to 3' direction.

*^b^* The restriction sites are underlined by bold letters.
