# Supplementary material for: High-Throughput Identification of Promoters and Screening of Highly Active Promoter-5′-UTR DNA Region with Different Characteristics from Bacillus thuringiensis
Source: PLoS One. 2013 May 10;8(5):e62960. doi: 10.1371/journal.pone.0062960 (PMC3651082; doi:10.1371/journal.pone.0062960)
Supplement: Table S4 — Highly active complex candidates selected in this study. (DOC) [file pone.0062960.s011.doc]

**Table S4 Highly active complex candidates selected in this study**

| Complex | ORF Code^a^ | ORF Function | Transcriptomics data of CT-43**^b^** | | | |
| --- | --- | --- | --- | --- | --- | --- |
|  |  |  | 7 h 9 h 13 h 22 h | | | |
| P*hj1* | CH0240 | co-chaperonin GroES | 6069 | 2721 | 21086 | 17 |
| P*hj2* | CH3483 | cold shock protein | 370299 | 512020 | 44963 | 0 |
| P*hj3* | CH4459 | 50S ribosomal protein L21 | 21230 | 19457 | 3605 | 387 |
| P*hj4* | CH4727 | DeoR family  transcriptional regulator | 33709 | 191333 | 6206 | 768 |
| P*hj5* | CH1410 | ferredoxin | 14259 | 6102 | 6494 | 0 |
| P*hj6* | pCT8252.6 | TasA protein | 59 | 163 | 2086 | 581 |
| P*hj7* | pCT281.042 | hypothetical protein | 19289 | 182232 | 18265 | 0 |
| P*hj8* | pCT14.11 | hypothetical protein | 737 | 218 | 4390 | 74 |
| P*hj9* | pCT6880.5 | hypothetical protein | 1385 | 1240 | 934 | 1898 |
| P*hj10* | pCT281.277 | pesticidal crystal protein Cry1Aa | 8 | 19 | 6262 | 479 |
| P*hj11* | CH1160 | spore coat protein Y | 0 | 0 | 18571 | 153 |
| P*hj12* | CH5430 | cell wall hydrolase CwlJ | 0 | 0 | 13940 | 0 |
| P*hj13* | CH5068 | hypothetical protein | 0 | 0 | 8337 | 0 |
| P*hj14* | CH4970 | phage protein | 0 | 0 | 19030 | 0 |
| P*hj15* | CH0446 | small, acid-soluble spore protein | 0 | 0 | 38954 | 0 |
| P*hj16* | CH3874 | calcium-transporting ATPase | 0 | 6 | 1073 | 0 |
| P*hj17* | CH4053 | hypothetical protein | 0 | 0 | 457 | 1340000 |
| P*hj18* | CH1986 | spore coat protein G | 0 | 0 | 78 | 230911 |
| P*hj19* | CH2337 | spore appendage protein | 0 | 0 | 0 | 97529 |
| P*hj20* | pCT281.285 | hypothetical protein | 0 | 0 | 35 | 9553 |

***^α^*** This column represented the number of the ORF downstream of the promoter in the chromosome (CH) or the plasmids (pCT) of CT-43. ***^b^*** The numerical value was the RPKM value (Reads Per Kilobase per Million mapped reads).
