# Supplementary material for: High-Throughput Identification of Promoters and Screening of Highly Active Promoter-5′-UTR DNA Region with Different Characteristics from Bacillus thuringiensis
Source: PLoS One. 2013 May 10;8(5):e62960. doi: 10.1371/journal.pone.0062960 (PMC3651082; doi:10.1371/journal.pone.0062960)
Supplement: Table S5 — Consensus sequences recognized by the σ-factor in the complex candidates. (DOC) [file pone.0062960.s012.doc]

**Table S5 Consensus sequences recognized by σ-factor in the complex candidates**

| Complex*^α^* | σ-factor | Consensus sequences*^b^* | Connection |
| --- | --- | --- | --- |
| **Complexes acting throughout the life-cycle** | | | |
| P*hj1* | σ^H^ | GAC**AGGAGAA**AGTTGTATGTGTAGC**GAAA**ACAACTTAGG | tandems |
|  | σ^K^ | GAA**AACA**ACTTAGGGTGTCCTTT**CATA**TTG**TA**ACATACGA |  |
|  | σ^A^ | TTAC**TTGCA**AAAGAAATTGAGATTATT**TATTAT**TATAAGTG |  |
| P*hj2* | σ^K^ | AAA**AAC**TTTACTTTTATTATAT**T**G**ATA**TGA**TA**TAGTATGTTT | tandems |
|  | σ^A^ | GGG**TTGCA**GTTTTTTAATATCTGGCT**TATTAT**TGTAAGGTG |  |
| P*hj3* | σ^A(1)^ | CAT**TTGAAA**TTTATTTCAAAGATGA**TATTAT**CGAAAAGT | tandems |
|  | σ^F^ | AA**GTAAA**AAAGAAATTGTAGAAC**GGAAAAAATA**GTTAGT |  |
|  | σ^A(2)^ | TCA**TTGACA**ATACTCAATGAATTATGG**TAACAT**CTTTATGT |  |
| P*hj4* | σ^A^ | AAT**TTGCA**GGAATTTGGCGTTTTACG**TAGAAT**TTTATGTG | overlapping |
|  | σ^H^ | GC**AGGAATTT**GGCGTTTTACGTA**GAATT**TTATGTGTTAGT |  |
| P*hj5* | σ^A(1)^ | AAG**TTGACA**CTCTAATTGATAAGCAT**TATCAT**TATAATTTT | σ^A(2)^ and σ^A(3)^  overlapping |
|  | σ^A(2)^ | ATG**TTGAAA**AGTTTCACAAACCGTTT**TACAAT**AAGGTTAT |  |
|  | σ^A(3)^ | GT**TTCACA**AACCGTTTTACAATAAGGT**TATAAT**TTTTATGG |  |
| P*hj6* | σ^A^ | TTG**TTTACA**TATTTTTAAAAATGAG**TATAAT**TGTATTAACG | tandems |
|  | σ^G^ | ACT**GTTATAA**CATTCTAACTAAACGT**CATA**ATAATACAG |  |
|  | σ^K^ | AAA**AGCA**AACAAACAAAACAAA**CATA**AAA**T**CACACTAT |  |
| P*hj7* | σ^A^ | ATA**TTGATA**TTTTTGGAAAATAAGG**TATTGT**TTACTTAATA | tandems |
|  | σ^E^ | TAAC**TAATATT**TGATATTAGTCTA**CATA**CTAAGAAA |  |
| P*hj8* | σ^A(1)^ | TTTA**TTTACA**AACCATATGGTTTTTGT**TATATT**AAATTTAG | overlapping |
|  | σ^A(2)^ | TTA**TTGAAA**AACCATATGTTTTTATT**TACAAA**CCATATG |  |
| P*hj9* | σ^A^ | TTG**TTGAAT**AGGATCAGTGTCATTTA**AATAAG**ATTGATTTC | tandems |
|  | σ^W^ | TGAG**GAAA**TGTCGAAATATATTAATT**CGTA**TTATTCTTAAA |  |
| **Complexes specifically induced in early-stationary phase** | | | |
| P*hj10* | σ^E^ | CTTTGT**GCATTT**TTTCATAAGATGAGT**CATATGTTT**TAAAT | overlapping |
|  | σ^K^ | TTAGTTGCACTTTGTGCATTTTTT**CATA**AGA**TG**AGTCATAT |  |
| P*hj11* | σ^E^ | TAAC**GTAATAT**CAACCATATCCCACT**CATA**TTG**TA**ATAGTG | / |
| P*hj12* | σ^E^ | GGTT**GTCATAT**CTGAGTCCTTTTTCT**CATA**CGTATGAACTA | / |
| P*hj13* | σ^E^ | GTGA**GCCTCT**ACTAATAAATATATG**ATA**CTG**T**TTAAATTT | / |
| P*hj14* | σ^H^ | TTAC**AGGAAT**CATTGTAGACAGTAAT**GAAT**AGTTATAAAC | tandems |
|  | σ^E^ | ACTA**TTGCAA**AAAGAGTTTATAG**T**A**ATA**ACA**TA**AAGAATT |  |
|  | σ^A^ | GAAT**TTCATA**ACAGCCTCCTTTTAAAC**TATATA**AGAAATG |  |
| P*hj15* | σ^G^ | TAA**TGAATGA**TGAAAATAAGCTAATTCATC**TTC**TTTCCATT | / |
| P*hj16* | σ^E^ | ATA**GCATTT**TTTTGGACGAGGCTGA**ATA**AGC**T**TTCAATAG | / |
| **Complexes specifically activated in mid-stationary phase** | | | |
| P*hj17* | σ^G^ | TCC**TGTATAT**ATCATCGTTTTATATA**AAAGTAAA**AAAGTGT | σ^G^ and σ^B^  overlapping |
|  | σ^B^ | TCAT**CGTTT**TATATAAAAGTAAAAAA**GTGTAA**AGGCTACC |  |
|  | σ^K^ | CCA**GTCA**CTCACTACCGAACTTA**CATA**GAT**TA**TAAGTGTG |  |
| P*hj18* | σ^K^ | CCTGT**TC**AGAAAGATGCCTACATA**CATATCATG**TTAAGGA | / |
| P*hj19* | σ^E^ | CTT**GCACTT**TTAAATGAGACAAG**CATA**TGT**T**GTAGTATA | tandems |
|  | σ^K^ | CGT**AACC**AAATCTATCAATCTAA**CATA**TTG**TA**TAAAAGT |  |
| P*hj20* | σ^K(1)^ | AG**GGCA**TACAATCCTCCTGTTT**CATA**AGC**TA**TTTTTCATTT | σ^K(1)^ and σ^G^ overlapping |
|  | σ^G^ | AGG**GCATA**CAATCCTCCTGTTTCAT**AAGCTAT**TTTTCATTT |  |
|  | σ^B^ | CGA**AGTGTTT**CATAAGACGGAATATT**GGTAAT**GTCTCTAT |  |
|  | σ^K(2)^ | CCA**ATCA**ATAAAATAATGTCATA**CATA**CAT**TA**GAAAAAAC |  |

***^α^***Here, the complexes’ names represent their promoter regions. ***^b^***The predicted consensus sequences recognized by σ-factor are underlined by bold letters.
